# Major vault protein is part of an extracellular cement material in the Atlantic salmon louse (*Lepeophtheirus* *salmonis*)

**DOI:** 10.1038/s41598-024-65683-0

**Published:** 2024-07-02

**Authors:** Malene Skuseth Slinning, Thaddaeus Mutugi Nthiga, Christiane Eichner, Syeda Khadija, Leonard H. Rome, Frank Nilsen, Michael Dondrup

**Affiliations:** 1 Sea Lice Research Centre (SLRC), Department of Biological Sciences, University of Bergen, Pb. 7803, 5020 Bergen, Norway; 2 SLRC, Computational Biology Unit (CBU), Department of Informatics, University of Bergen, Pb. 7803, 5020 Bergen, Norway; 3 Department of Biological Chemistry, David Geffen School of Medicine and the California NanoSystems Institute, University of California Los Angeles, Los Angeles, CA 90095 USA

**Keywords:** Salmon louse, Vault complex, Major vault protein (MVP), Cement gland, Secretory protein, Proteomics, Phylogenetics, Organelles, Parasitic infection, Zoology

## Abstract

Major vault protein (MVP) is the main component of the vault complex, which is a highly conserved ribonucleoprotein complex found in most eukaryotic organisms. MVP or vaults have previously been found to be overexpressed in multidrug-resistant cancer cells and implicated in various cellular processes such as cell signaling and innate immunity. The precise function of MVP is, however, poorly understood and its expression and probable function in lower eukaryotes are not well characterized. In this study, we report that the Atlantic salmon louse expresses three full-length MVP paralogues (LsMVP1-3). Furthermore, we extended our search and identified MVP orthologues in several other ecdysozoan species. LsMVPs were shown to be expressed in various tissues at both transcript and protein levels. In addition, evidence for LsMVP to assemble into vaults was demonstrated by performing differential centrifugation. LsMVP was found to be highly expressed in cement, an extracellular material produced by a pair of cement glands in the adult female salmon louse. Cement is important for the formation of egg strings that serve as protective coats for developing embryos. Our results imply a possible novel function of LsMVP as a secretory cement protein. LsMVP may play a role in structural or reproductive functions, although this has to be further investigated.

## Introduction

Vaults are highly conserved ribonucleoprotein complexes present in the majority of eukaryotic organisms^1^. The vault complex was discovered in the mid-1980s and has since been extensively studied^2^. The vault complex is about three times the size of a ribosome with a molecular mass of 13 MDa and dimensions of 67 nm × 40 nm × 40 nm^3,4^. It has a characteristic hollow barrel-like structure, and the mammalian vault complex is composed of 78 copies of the major vault protein (MVP, 100 kDa), ~ 2 copies of telomerase-associated protein 1 (TEP1, 240 kDa), ~ 8 copies of vault poly (ADP-ribose) polymerase (vPARP, 193 kDa), and at least 6 copies of small untranslated vault RNAs (vRNAs)^2,3,5–7^. MVP is the main component of the vault complex, accounting for more than 70% of its mass, and is also the only component essential for the formation of vaults^8^. It has also been proposed that vaults are assembled by polyribosome templating, in which a single *MVP* mRNA copy is translated on a polyribosome and the MVP monomers are directly assembled into a vault particle^9^.

The vault complex is highly abundant in most tissues and cell types^1,10–13^, however, no clear function has been identified so far. Vaults have been suggested to be involved in several cellular functions such as nuclear-cytoplasmic transport, multidrug resistance, cell signaling, and innate immunity^12,14–17^. However, knockout studies of different vault components in mice did not show any phenotype, indicating that vaults do not have a critical cellular function^18–20^. A reason for the lack of understanding of its function could be due to the absence of vaults in some of the most important model organisms such as fruit fly (*Drosophila* *melanogaster*), yeast (*Saccharomyces* *cerevisiae*), roundworm (*Caenorhabditis* *elegans*), and plant (*Arabidopsis* *thaliana*)^21^. Although, vaults have been identified and characterized in other eukaryotes such as amphibians, avians, fish, sea urchin (*Strongylocentrotus* *purpuratus*) and slime mold (*Dictyostelium* *discoideum*)^1,11,22–24^. Daly et al.^25^ studied the extension of MVP across the Eukaryote kingdom and proposed that it was likely already present in the last Eukaryote common ancestor (LECA) but also proposed that the gene might have been lost in major clades such as Arthropods and Fungi.

The salmon louse (*Lepeophtheirus* *salmonis*) is a marine crustacean ectoparasite of salmonids in the Northern Hemisphere. The salmon louse has a direct life cycle that consists of eight distinct developmental stages, separated by a molt^26^. The first stages of the salmon louse life cycle are planktonic, comprising of nauplius I, nauplius II, and the infective copepodid stage. During the copepodid stage, the salmon louse must attach to a fish host. Once attached, the salmon louse feeds on the host's skin, mucus, and blood, developing through the parasitic stages: chalimus I, II, pre-adult I, II, and ultimately reaching sexual maturity in the adult stage^26,27^. Upon fertilization, the female salmon louse produces a pair of egg strings containing the developing embryos that eventually will hatch to nauplii larvae. During the extrusion of the egg strings, the embryos are covered with a cement material which forms a protective layer for the embryos, and whose molecular composition has been previously studied^28^.

Recently, genomes of the Atlantic and Pacific subspecies of *L.* *salmonis* were sequenced and several interesting gene losses and expansions were identified^29,30^. The salmon louse was shown to lack genes for peroxisome biogenesis and heme degradation and to have a small number of detoxification genes^29^. However, among the few expanded gene families in the genome, were genes related to the vault ribonucleoprotein complex.

In the present study, we identified and characterized MVP in the Atlantic salmon louse. MVP orthologues were shown to be present in the majority of ecdysozoan lineages, with three full-length MVP paralogues (LsMvp1-3) identified in the Atlantic salmon louse genome. While *LsMvp* transcripts were expressed in various tissues in both male and female salmon lice, LsMVP protein was predominantly enriched in the cement, a sticky egg string-forming material that is synthesized and secreted by a pair of cement glands in the female salmon louse.

## Materials and methods

### Bioinformatics and phylogenetic analyses

Gene models were obtained by aligning transcript sequences to Atlantic and Pacific salmon louse genome assemblies^29,30^ with GMAP^31^ and exonerate^32^.

Candidate orthologues of MVP were detected by an iterative process (1) BlastP^33^ online searches against GenBank using the LsMVP sequences as templates, (2) from unannotated whole genome shotgun (WGS) and transcriptome shotgun (TS) assemblies downloaded from GenBank, and (3) from selected TS raw data downloaded from the Sequence Read Archive (SRA). WGS draft genomes were first scanned for MVP orthologues by TBlastN. Then, orthologous coding sequences (CDS) were extracted from contigs with significant hits using exonerate in protein2genome mode, using a selected template from step (1). In case of multiple hits, we selected the hit with the highest alignment score. The derived CDS was then translated using EMBOSS getOrf^34^ in all six reading-frames and the longest open-reading frame was selected as candidate. Unassembled TS raw data was assembled with Trinity. All TS assemblies were then scanned for MVP orthologues with TBlastN. Best hitting contigs were extracted and translated in all six reading-frames with getOrf. The resulting candidates were subjected to BlastP and InterProScan searches to further confirm their identity. E-value cutoff was set to 1e−6 for all Blast searches. Protein 3D structures for all candidates were predicted in AlphaFold2^35^ in monomer mode through the Sigma2 NRIS high-performance computing infrastructure and the EuroHPC supercomputer LUMI. Top-ranked AlphaFold2 models (ranked_0.pdb) were structurally aligned to Chain A of the experimental structure (PDB: 4V60)^3^ as reference using TM-align^36^. Protein structures were visualized with Chimera^37^.

Multiple-sequence alignment (MSA) of protein sequences was computed in Muscle^38^ with default settings and inspected and end-clipped to the first and last conserved column in Jalview^39^. Maximum-likelihood phylogenies were generated in IQ-TREE 2.2.3 using ModelFinder (Best-fit model according to BIC: Q.insect + R7) and 1000 ultra-fast bootstrap replicates^40,41^. Bayesian phylogenies were computed with MrBayes’ Markov-Chain-Monte-Carlo sampling^42,43^ over 5 million generations in mixed mode (model with highest posterior probability: Rtrev) using 4 parallel chains and 4 gamma categories and allowing invariant sites. Trees were visualized with FigTree^44^. For gene loss analysis, we downloaded all genome assemblies for the representative taxonomic groups using the NCBI datasets command line tool, created a Blast database per taxon and scanned for MVP orthologues with TBlastN using a selection of MVP sequences as templates.

### Antibodies

Primary antibodies used in this study were anti-human MVP/LRP (Proteintech, Cat# 16478-1-AP), anti-GAPDH (Sigma-Aldrich, Cat# G9545), anti-Myc tag (9B11) (cell signaling, Cat# 2276), and anti-GFP (Abcam, Cat# ab290). To test the specificity of the anti-human MVP antibody for LsMVP, both human and salmon louse LsMVP2 were in-vitro translated using the TNT T7 Reticulocyte Lysate System (Promega, Cat# L1170) according to the manufacturer’s protocol and subsequently, 1 μl of the in vitro-translated proteins were used for the antibody testing by western blotting.

### Ethical statement

All experiments were conducted in accordance with relevant guidelines and regulations. The study was performed with approval granted from the Ethic committee of Norwegian Food Safety Authority (Permit ID: 8589 and 26020). All methods are reported in accordance with ARRIVE guidelines (https://arriveguidelines.org).

### Animal cultivation

*Lepeophtheirus* *salmonis* *salmonis* (LsGulen) strain was maintained on farmed Atlantic salmon (*Salmo* *salar*) in the laboratory as described by Hamre et al.^45^. Atlantic salmon were kept in seawater with a salinity of 34.5 ppt at ~ 9 °C and hand fed with a commercial diet. Egg strings were hatched in a flowthrough single well system, where nauplii and copepods were kept^45^.

### Sampling and tissue dissection of salmon lice

Tissues were dissected from adult female salmon lice. For ovaries, the ventral exoskeleton was removed, while the subcuticular tissue was dissected by cutting the outer edges of the cephalothorax. Intestinal tissue was retrieved from the abdomen of the salmon louse (see Fig. 4a). Cement or cement glands were isolated as described by Borchel et al.^28^. cDNA of adult female salmon lice at different timepoints of the egg string cycle were kindly provided by Dr. Andreas Borchel and sampled as described by Borchel et al.^28^. Samples for qPCR measurements were put directly on RNAlater (Thermo Fisher Scientific) at 4 °C overnight and then at − 20 °C until use for RNA isolation. Samples for western blot analysis were added directly to the SDS-sample buffer (0.1 M Tris pH 6.8, 2% SDS, 10% glycerol, 0.4 M DTT, bromophenol blue), crushed with a pestle and boiled at 99 °C for 5 min or until dissolved (cement). Samples were used directly for SDS-PAGE and western blot analysis or stored at − 20 °C.

### RACE-PCR, cloning, and sequencing

Full-length cDNA of *LsMvp1* (EMLSAG00000005145), *LsMvp2* (EMLSAG00000005383), *LsMvp3* (EMLSAG00000002586), and *LsTep1* (EMLSAG00000005384) from LiceBase/ENSEMBL was determined by rapid amplification of cDNA ends (RACE) PCR. SMARTER™ RACE cDNA amplification kit (Clontech) was used to generate 5′ and 3′ RACE-ready cDNA following the manufacturers protocol. Advantage^®^ 2 PCR enzyme system (Clontech) was further used for the amplification of the cDNA target. PCR products were cloned using TOPO^®^ TA Cloning^®^ for sequencing (Invitrogen) and One Shot TOP10 Chemically Competent *Escherichia* *coli* cells. Clones were subsequently amplified by PCR using GoTaq^®^ DNA Polymerase (Promega) with M13 forward and reverse primers. ExoCleanUp FAST PCR reagent (VWR) was used for PCR clean-up followed by sequencing using the BigDye Terminator v.3.1 cycle sequencing kit (Applied Biosystems). Samples were sequenced at the University of Bergen (UoB) sequencing facility on an ABI prism 7700 automated sequencing machine. Additional primers were used to sequence CDS of *LsTep1* (Primers are listed in Supplementary Table S1). Sequences were analyzed using Vector NTI (Invitrogen) or the online-based tool Benchling (https://benchling.com/) and the protein molecular weight was predicted using Expasy ProtParam tool^46^.

### RNA isolation and cDNA synthesis

One whole adult salmon louse, or salmon louse tissues from 3 to 6 animals were homogenized in Tri Reagent (Sigma) using a TissueLyser II bead mill (Qiagen, Hilden, Germany) for 5 min and 30 Hz with 5 mm stainless steel beads or 1.4 mm zirconium oxide beads (Qiagen). Chloroform separation was performed after homogenization, following RNA isolation using Direct-Zol RNA micro or mini plus kit (Zymo Research) for tissues, or adult females, respectively. Samples were treated with DNase digestion on column according to the manufacturer’s protocol. RNA was stored at − 80 °C or used directly in cDNA synthesis.

cDNA synthesis for qPCR measurements was performed using AffinityScript QPCR cDNA Synthesis kit (Agilent Technologies) following the protocol with 200 ng RNA, 10 ng/µl of oligo (dT) primer and 5 ng/µl random primers. The reaction was incubated at 25 °C for 5 min, 42 °C for 15 min, and 95 °C for 5 min. The cDNA was diluted ten times and stored at − 20 °C until use. qScript^®^ cDNA SuperMix (Quantabio) was used for cDNA synthesis for standard PCR according to the manufacturer`s protocol with 1 µg total RNA.

### Quantitative real-time PCR

Gene expression analysis was performed on QuantStudio 3 qPCR machine (Applied Biosystems) using PowerUp™ SYBR™ Green Master Mix (Applied Biosystems), 0.5 µM of forward and reverse primer, and 2 µl cDNA in a final volume of 10 µl. PCR cycling profile was run with an initiation at 50 °C for 2 min, holding at 95 °C for 2 min followed by 40 cycles of 95 °C for 15 s and 60 °C for 1 min. No template control (NTC) and minus reverse transcriptase (− RT) was run for all assays. All samples were run in duplicates. Relative gene expression of different salmon louse tissues was quantified by E^−∆Ct^^47^ using the reference genes elongation factor 1α (*EF1α*) and Adenine Nucleotide Translocator 3 (*ADT3*). The relative gene expression of adult salmon lice at different timepoints during egg string development was calculated relative to the reference gene *EF1α* and *ADT3* and normalized to the average expression of all samples for each gene product (E^−∆∆Ct^). Primer efficiencies were calculated from a standard curve with five points using twofold dilutions. Primer sequences with additional information are shown in Supplementary Table S2 and Supplementary Fig. S7.

### In situ hybridization

Adult female salmon lice were fixed in 4% paraformaldehyde/phosphate buffered saline (PBS) at 4 °C overnight and processed with the Histokinette 2000 (Reichert-Jung) where the samples were washed in PBS and dehydrated in a graded ethanol series and embedded in paraffin wax. 4 µM horizontal sections were cut using a Leica RM 225 microtome (Leica Microsystems). Template for the sense- and anti-sense probes were generated by PCR using cDNA and primers with T7 promoter incorporated into either the reverse or forward primer (see Supplementary Table S1). Digoxigenin (DIG) labeled RNA probes were then synthesized using DIG RNA labeling kit (Rocher). In situ hybridization was performed following Dalvin et al.^48^, with some modifications as previously described by Tröße et al.^49^. Proteinase K digestion was increased to 18 min. Sections were incubated with either anti-sense or sense probes (674 bp) at a concentration of 22 ng/µl targeting *LsMvp2* and *LsMvp3*. The probes have 75% identity to the LsMvp1 nucleotide sequence as well.A signal was developed using BCIP^®^/NBT liquid Substrate System (Sigma-Aldrich). ImmunoHistoMount™ (Sigma-Aldrich) was used for mounting and images were acquired with Zeiss Axio Scope A.1 microscope. A sense probe was used as a negative control.

### Immunohistochemistry

Paraffin sections (4 µM) were baked at 65 °C for 30 min and incubated in Histoclear for 2 × 10 min. Sections were further washed in a decreasing ethanol series (2 × 100%, 95%, 80%, 50%) for 3 min each and stored in MilliQ water until antigen retrieval. Antigen retrieval was performed in sodium citrate buffer (10 mM Tri-Sodium Citrate dihydrate, 0,05% Tween 20, pH 6.0) in a water bath at 95 °C for 30 min. Sections were then left to cool for 30 min at room temperature and washed three times for 5 min in TBS-T (50 mM Tris, 150 mM NaCl, 0,05% Tween 20, pH 7.6). Sections were blocked overnight with 5% BSA in TBS-T in a humidified chamber at 4 °C. Sections were incubated with MVP/LRP Polyclonal antibody (Proteintech) diluted 1:200 in 1% BSA in TBS-T for 1 h at room temperature and incubated with secondary antibody Anti-Rabbit IgG (whole molecule)-AP (Sigma-Aldrich) diluted 1:100 in 1% BSA in TBS-T at room temperature for 1 h. Three washes of 5 min with TBS-T were performed after each antibody incubation. Sections were then incubated in processing buffer (100 mM Tris-NaCl, 50 mM MgCl_2_, pH 9.5) for 10 min. A signal was developed using BCIP^®^/NBT liquid Substrate System (Sigma-Aldrich) before sections were washed with MilliQ water and mounted with ImmunoHistoMount™ (Sigma-Aldrich). Images were acquired with Zeiss Axio Scope A.1 microscope. In the negative control the primary antisera was omitted. For DAPI staining of the cement gland, sections were mounted with Fluoroshield™ with DAPI histology mounting medium (Sigma-Aldrich). Fluorescence images were acquired with Leica Fluorescence microscope DMI 6000 B.

### Isolation of microsomal pellet

26 adult female salmon lice were homogenized in a buffer containing 50 mM Tris (pH 7.4), 75 mM NaCl, 1.5 mM MgCl_2_, 1 mM DTT, and 1 × complete protease inhibitor cocktail using a Dounce homogenizer. The sample was spun down at 4500 × g for 15 min before the supernatant was spun down at 20,000 × g for 20 min. Finally, the supernatant was spun down at 30,000 rpm (~ 111,000 × g, SW41 Ti rotor) for 1 h. The pellet was resuspended in 2 × SDS-sample buffer, while 4 × SDS sample buffer was added to the supernatant. Samples were loaded onto a 4–20% SDS-gel and run at 28 mA for 45–60 min. The gel was transferred to a 0.2 µm PVDF membrane (Bio Rad, Cat# 1704156) on a Trans-Blot^®^ Turbo™ Transfer System machine (Bio-Rad) followed by antibody incubations as described for western blot analysis.

### Western blot analysis

Protein extracts were loaded onto a 4–20% or 10% SDS gel (Mini-PROTEAN TGX Stain-Free Precast Gels, Bio-Rad) and run at 28 mA for 45–60 min. The gel was then soaked in transfer buffer (300 mM Tris, 300 mM glycine, 0.1% SDS, 20% MeOH) and transferred to 0.2 µm nitrocellulose membrane on a Trans-Blot^®^ Turbo™ Transfer System machine (Bio-Rad). Blots were incubated with MVP/LRP Polyclonal antibody (Proteintech) diluted 1:1000 in 5% (w/v) nonfat milk in TBS-T overnight at 4 °C and subsequently incubated with HRP conjugated secondary antibody in 2.5% (w/v) nonfat milk in TBS-T. Blots were washed three times for 5 min with TBS-T after each antibody incubation. Immunoblot bands were detected using SuperSignal™ West Pico PLUS Chemiluminescent Substrate (Thermo Fisher Scientific) and imaged with ChemiDoc XRS+™.

### Proteomics analysis

For mass-spectrometry analysis, an SDS-gel was stained with QC Colloidal Coomassie stain (Bio-Rad) and destained with MilliQ water. A band of interest was then cut with a sterile scalpel and sent to the Proteomics Unit at the University of Bergen (PROBE) for analysis. In brief, gel pieces were washed before subjected to reduction with DTT, alkylation with iodoacetamide and digestion with trypsin. About 0.5 µg tryptic peptides were dissolved in 2% acetonitrile (ACN) and 0.5% formic acid and injected into an Ultimate 3000 RSLC system (Thermo Scientific, Sunnyvale, California, USA) connected online to an Orbitrap Eclipse mass spectrometer (Thermo Scientific, Bremen, Germany) equipped with EASY-spray nano-electrospray ion source (Thermo Scientific). The sample was desalted on a pre-column (Acclaim PepMap 100, 2 cm × 75 µm ID nanoViper column, packed with 3 µm C18 beads) at a flow rate of 5 µl/min for 5 min with 0.1% trifluoroacetic acid.

Peptides were separated during biphasic ACN gradient from two nanoflow UPLC pumps (flow rate 200 nl/min) on a 25 cm analytical column (PepMap RSLC, 25 cm × 75 µm ID. EASY-spray column, packed with 2 µm C18 beads). Solvent A was 0.1% FA (vol/vol) in water and solvent B was 100% ACN. The gradient composition was 5% B during trapping (5 min) followed by 5–7% B over 1 min, 7–20% B for the next 64 min, 20–32% B over 17 min, and 32–85% B over 3 min. Elution of very hydrophobic peptides and conditioning of the column were performed for 10 min isocratic elution with 85% B and 15 min isocratic conditioning with 5% B respectively. Instrument control was through Thermo Scientific SII for Xcalibur 1.6. Peptides eluted from the column were detected in the Orbitrap Eclipse Mass Spectrometer with FAIMS enabled using three compensation voltages (CVs), − 50 and − 70. During each CV, the mass spectrometer was operated in the DDA-mode (data-dependent-acquisition) to automatically switch between one full scan MS and MS/MS acquisition. The spray and ion-source parameters were as follows: Ion spray voltage = 1800 V, no sheath and auxiliary gas flow, and capillary temperature = 275 °C.

Reference database was obtained from LiceBase/ENSEMBL, and data search was performed using Proteome Discoverer (Thermo Fisher Scientific).

### RNA interference

RNA interference (RNAi) was carried out as described in Dalvin et al.^50^. Double stranded RNA (dsRNA) was synthesized using MEGAscripts^®^ RNAi Kit (Ambion, Austin, TX, USA) according to the manufactures protocol. DsRNA fragments targeting (*LsMvp1*, 576 bp), and (*LsMvp2/3*, 445 bp) were synthesized from amplified PCR products using cDNA with primers listed in Supplementary Table S1. Cod trypsin (CYP) sequence was used as a negative control. Pre-adult II female salmon lice were injected with a mixture of the two dsRNA with a final concentration of 600 ng/µl for *LsMvp1* and 600 ng/µl for *LsMvp2/3*, or with 600 ng/µl control dsRNA. Five μl of saturated trypan blue was added to 50 μl of dsRNA solution. An additional control group with lice injected with diluted trypan blue solution only was run in experiment 2. For each group (control- and knock-down-groups in both experiments) 30 lice were injected, let to recover in sea water and then being distributed to 3 fish for each group, assigned to individual fish tanks. After 34 (experiment 1) or 41 (experiment 2) days, the salmon lice were sampled, photographed, and put on RNAlater for qPCR measurements or in Karnovsky`s fixative for histological samples.

## Results

### Sequence analysis of vault-related genes

**Figure 1:**
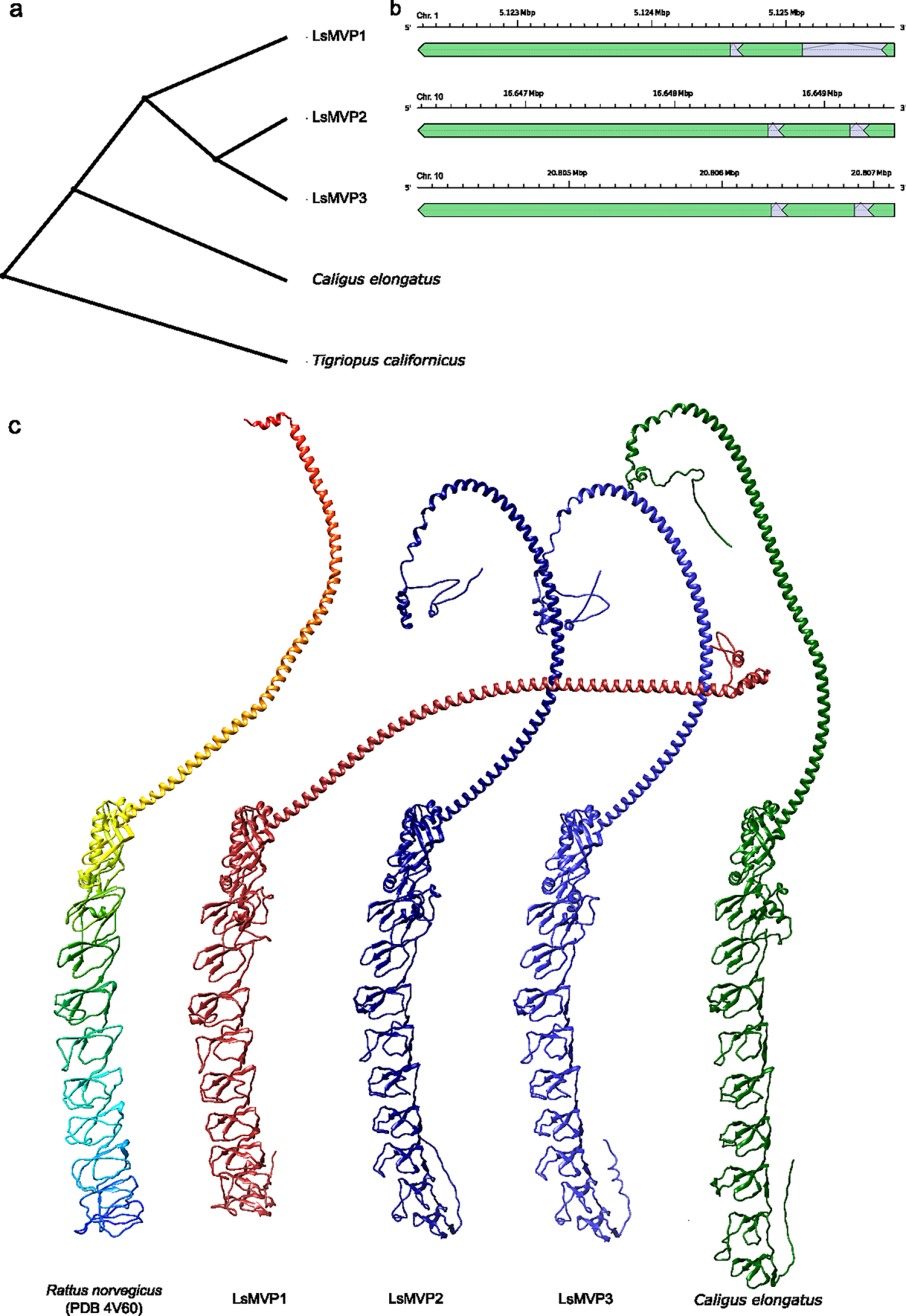
Sequence analysis and 3D-structure modelling of LsMVP orthologues. (**a**) ML cladogram of the three full-length LsMVP paralogues and MVP orthologues identified in *C.* *elongatus* and *T.* *californicus* with the tree rooted on *T.* *californicus.* (**b**) Gene models were derived from alignment of the RACE consensus transcript sequences to the chromosome level genome assembly of the Pacific salmon louse (GCF_016086655.3), exons plotted in green, introns indicated by arcs. Scale bars represent chromosomal location. (**c**) Protein tertiary structures of LsMVP and *C.* *elangatus* orthologues as predicted by AlphaFold2 from the hypothetical protein sequences and the experimental structure for comparison (PDB 4V60, Chain A).

Several vault-related genes were previously identified in the salmon louse genome^29^. In the present study, transcripts of *LsMvp1* (EMLSAG00000005145, GenBank ID: PP449126), *LsMvp2* (EMLSAG00000005383, GenBank ID: PP449127), *LsMvp3* (EMSALG00000002586, GenBank ID: PP449128), and *LsTep1* (EMLSAG00000005384, GenBank ID: PP449129), were validated by RACE-sequencing. LsMvp1 localizes to chromosome 1 while LsMvp2 and LsMvp3 localize to chromosome 10 and their transcript sequence consists of three exons each (Fig. 1b).

LsMVP1 (96.2 kDa) encodes a protein of 859 aa. LsMVP2 (97.1 kDa) and LsMVP3 (97.6 kDa) encode proteins of 867 aa and 872 aa, respectively, and were shown to have a high sequence identity of 95.2%. LsMVP1 had a sequence identity of 80.5% and 80.4% for LsMVP2 and LsMVP3, respectively. Rooted phylogenetic trees show that the earliest gene diversification was likely between LsMvp1 and LsMvp2/3 (Fig. 1a). Ab initio modelling of the protein tertiary structure by AlphaFold2 results in models highly similar to the experimental structure of rat MVP (TM-score: 0.72–0.74) (Fig. 1c).

A Tep1 homologue was identified in the salmon louse genome with a protein length of 2427 aa (280 kDa). This LsTep1 gene was found to co-localize with LsMvp2 on the opposite DNA strand. Another possible truncated version of a LsTep1 homologue was identified on the opposite DNA strand of LsMvp3 in the genome, but we did not manage to retrieve the 3′end sequence using RACE-PCR. The other components found to be associated with mammalian vaults; vPARP and vRNA could not be identified in the salmon louse genome.

### MVP orthologues are extant in the majority of ecdysozoan lineages

**Figure 2:**
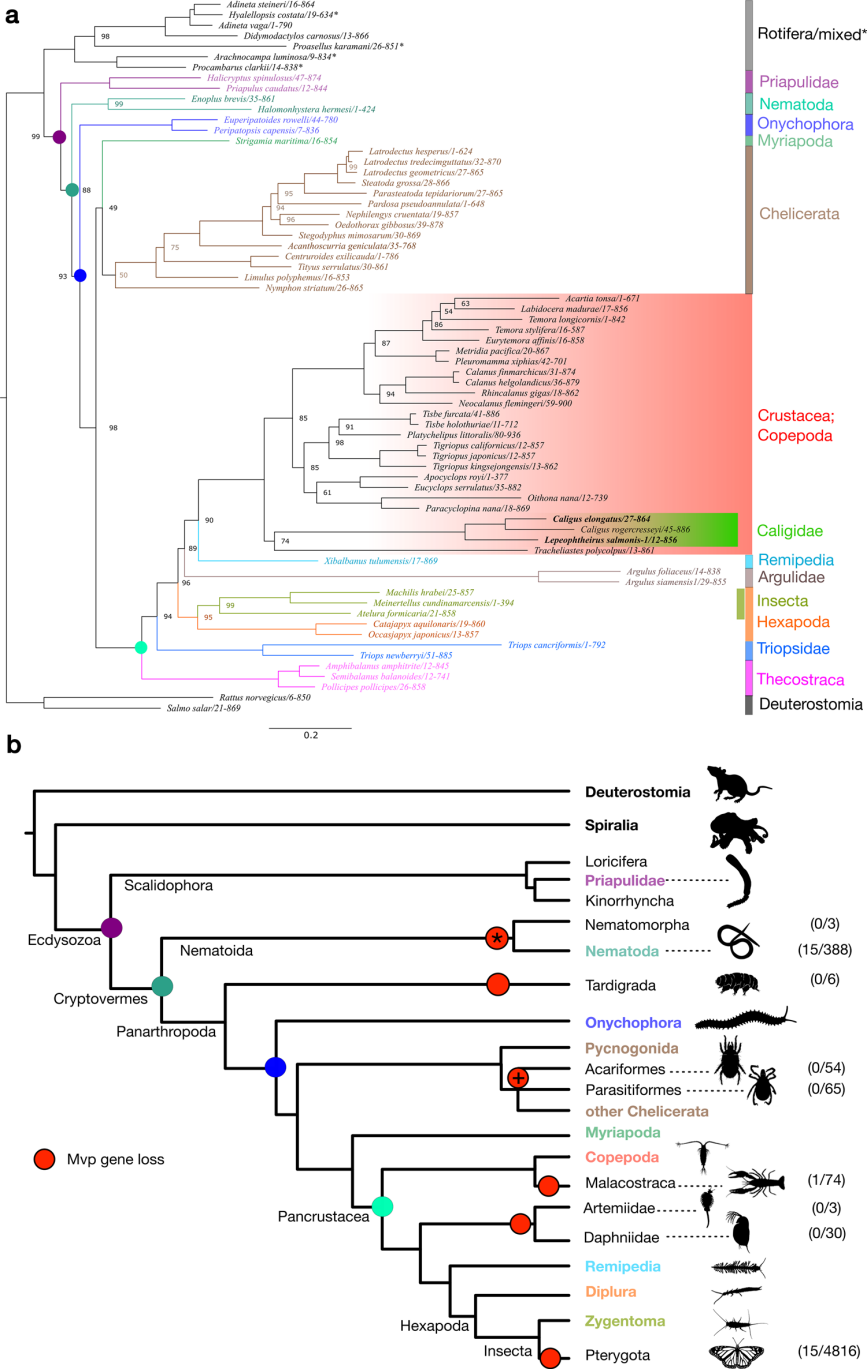
Phylogenetic analysis and proposed gene loss events for LsMVP orthologues in Ecdysozoa. (**a**) Maximum-likelihood phylogenetic tree of selected MVP orthologues, major monophyletic clades are annotated with colored bars, taxa in bold have experimental evidence in this study. Node support in % is given for all nodes with bootstrap support < 100%, scale-bar represents substitutions per site. Taxa marked with an asterisk indicate likely presence of contaminants. The tree was rooted using the Deuterostomia as outgroup. The MSA of orthologous MVP sequences used to construct the phylogenetic tree are given in Supplementary File S2. (**b**) Likely parsimonious gene loss events (red circles) of Mvp projected on a consensus topology of Ecdysozoa following Howard et al.^51^. Taxa in bold contain MVP-orthologues and are colored in the same way as in (**a**). In parenthesis: number of genome records with significant (e-value < 1E−6) Blast hit/total number of records scanned. Gene loss marked with *: Possible mosaic distribution and presence of orthologues in a few taxa; + : Following Lozano-Fernandez et al.^52^ we assume monophyly of Acari (Acariformes + Parasitiformes) and thereby assign a single ancestral gene loss to this group. Colored nodes reference the same major diversification events in both trees (**a**) and (**b**). Pictograms were downloaded from https://phylopic.org.

We performed an extensive database search for MVP orthologues in GenBank, reference and draft genomes and transcriptome assemblies using the salmon louse protein sequences as templates. We found significant sequence hits in 41 ecdysozoan taxa, comprising such diverse groups as Crustacea and Chelicerata. In addition, we recovered several sequences from groups previously unknown to have MVP orthologues, namely two marine nematodes (*Enoplus* *brevis*, *Halomonhystera* *hermesi*), hexapod springtails (*Catajapyx,* *Ocasjapyx* *sp.*), and basal insects of the orders Archaeognatha and Zygentoma (Fig. 2a, Supplementary File S2). Phylogenetic analyses of the orthologous sequences group similar taxa and reproduce accepted monophyletic clades and diversification events such as Ecdysozoa, Panarthropoda, and Pancrustacea with varying bootstrap support (Fig. 2a). Within the Copepoda, grouping of taxa supports the four orders Calanoida, Cyclopoida, Harpacticoida, and Siphonostomatoida, which includes the salmon louse sequence, with signs of accelerated evolution in the Siphostomatoida group.

While MVP is present in the majority of clades, gene loss is also pervasive within the Ecdysozoa (Fig. 2b). When mapping possible gene loss events to a recent ecdysozoan phylogeny^51^, six or seven independent gene loss events appear as a conservative estimate. We did not find any credible MVP orthologue within the Tardigrades, Malacostraca, and the crustacean groups containing the families *Daphnia* *and* *Artemia*. We reject several sequences from transcriptomes of predominantly cave-dwelling isopods and an insect in our dataset that forms a clade with rotifers as likely contaminated.

The winged insects (Pterygota) form the most densely covered arthropod group with at least one gene loss event. Within 4816 assemblies we retrieved only 15 assemblies, predominantly from recent wild isolates, with significant Blast hits to MVP. We extracted two nearly identical full-length MVP orthologues from two different species (*Ecitophya* *simulans* and *Ecitomorpha* *arachnoides*). When comparing them to a large panel of MVP sequences, however, they were more similar to trypanosomatid protozoan parasites than the closest insect sequences (67.87% and 46.12% identity, respectively). We therefore reject these sequences as likely of symbiotic origin. The presence of MVP orthologues in the sister groups of terrestrial insects and hexapods suggests that the gene loss is ancestral to Pterygota but not all insects. In contrast to Pterygota, orthologous sequences recovered from nematodes form a plausible clade between Priapulidae and Onychophora.

### LsMVP is specifically recognized by the anti-human MVP antibody

To clearly identify and characterize salmon louse MVP protein, the commercially available anti-human MVP antibody (Proteintech, 16478-1-AP) was tested for the detection of salmon louse MVP by western blotting using extracts from human AU565 cells as positive control. A single band of approximately 100 kDa that corresponded with the human MVP was recognized by the antibody in the extracts from female salmon louse (see Supplementary Fig. S1a), suggesting that the antibody against human MVP cross-reacted with LsMVP. The antibody also recognized both human and salmon louse (LsMVP2) in vitro-translated GFP- and Myc tagged MVP (See Supplementary Fig. S1b,c), further indicating its specificity for LsMVP. An alignment of LsMVPs and the peptide sequence of the antibody’s immunogen showed that LsMVP1 had 49% identity while LsMVP2 and LsMVP3 shared 54% identity (see Supplementary Fig. S2). Consequently, we proceeded to characterize LsMVP using the antibody.

### mRNA and protein localization of LsMVP

**Figure 3:**
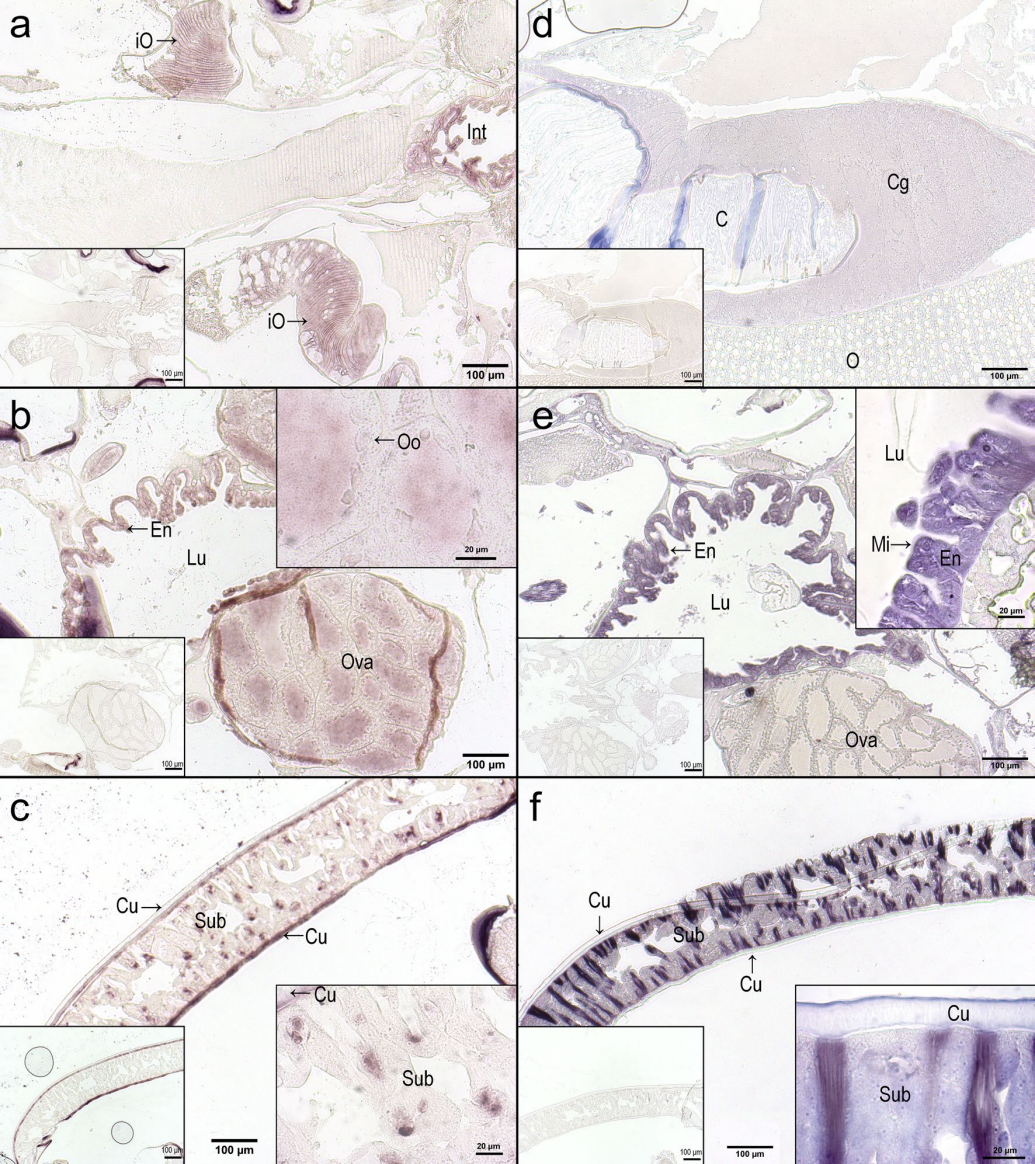
mRNA and protein localization of LsMVP. In situ hybridization (**a**–**c**) and immunohistochemistry (**d**–**f**) were performed to localize LsMVP mRNA and protein expression in the Atlantic salmon louse. *LsMvp* mRNA expression was localized to (**a**) immature oocytes, (**b**) intestine and ovaries, and (**c**) the subcuticular tissue. LsMVP protein was localized to (**d**) the cement gland, (**e**) intestine, and (**f**) the subcuticular tissue. Sense probe (in situ hybridization) or secondary antibody only (immunohistochemistry) were used as negative controls and did not give any signals (left corner boxed images). *Int* intestine, *iO* immature oocyte, *Cu* cuticula, *Sub* subcuticular tissue, *Ova* Ovarium, *Oo* oogonia, *En* enterocytes, *Lu* gut lumen, *Mi* microvilli, *C* cement, *Cg* cement gland, *O* oocytes.

Localization of LsMVP transcripts and proteins was determined by in situ hybridization and immunohistochemistry, respectively. LsMVP transcripts and proteins were both localized to the subcuticular tissue (Fig. 3c,f) and the intestine (Fig. 3b,e) of female and male salmon lice (Fig. 3 and Supplementary Fig. S3). *LsMvp* transcripts were also localized to the ovaries (Fig. 3b) and immature oocytes (Fig. 3a) of the adult female salmon louse. A faint LsMVP protein signal was observed in the cement gland (Fig. 3d), but the corresponding transcript was not detected during in situ hybridization.

### Gene and protein expression analysis of LsMVP

**Figure 4:**
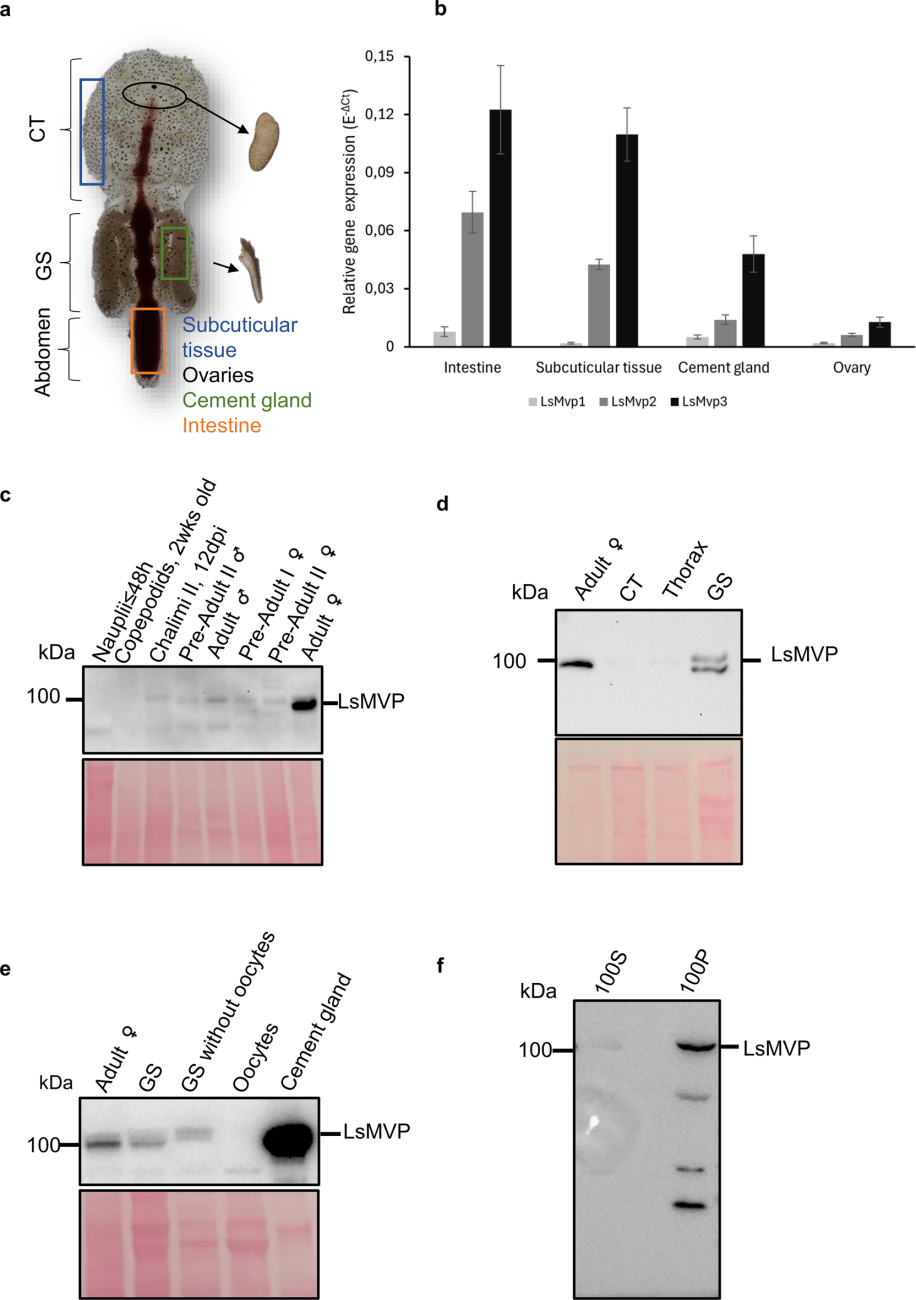
Gene and protein expression analyses of LsMVP. (**a**) Salmon louse anatomy. A schematic showing the different tissues that were dissected for gene and protein expression analyses. (**b**) Relative gene expression analysis (E^−ΔCt^ ± s.d.) by qPCR was performed on different salmon louse tissues (N = 3–4) showing a high expression in the intestine and subcuticular tissue compared to the cement glands and ovaries. (**c**) A cropped western blot showing analysis of the different life stages of the salmon louse showed a high protein abundance of LsMVP in the adult female salmon louse compared to the other life stages. (**d**) A cropped western blot analysis shows that LsMVP is highly expressed in the genital segment of the adult female salmon louse compared to the cephalothorax. (**e**) A western blot analysis showing protein abundances of LsMVP in different tissues of the genital segment in the adult female salmon louse. The cement gland was shown to have the highest amount of protein compared to oocytes. (**f**) A Western blot analysis after a differential centrifugation, identified LsMVP in the pellet after ~ 100,000 × g centrifugation (100P) compared to the supernatant (100S), suggesting that LsMVP is likely to assemble into vaults. *CT* Cephalothorax, *GS* Genital segment, *Dpi* Days post-infection. Original gel blot images are shown in Supplementary Fig. S8.

The expression level of *LsMvp* transcripts in the tissues that stained positively for LsMVP mRNA and protein (Fig. 3) was further determined by quantitative real-time PCR. Relative *LsMvp* gene expression analysis showed enrichment of the transcripts in the subcuticular tissue and intestine, while expressed at lower levels in the ovaries and cement glands of the adult female salmon louse (Fig. 4b). Moreover, we characterized the protein abundances of LsMVP in the salmon louse using western blot analysis. Protein analysis in different life stages of the salmon louse showed that the adult female salmon louse had the highest protein abundance compared to the chalimus, pre-adult, and adult male stages (Fig. 4c). The protein in the nauplii and copepodid stages was, however, not detected. Protein analysis of different body parts of the female salmon louse showed that most of the protein was present in the genital segment, while presence in the cephalothorax section was barely detectable (Fig. 4d), suggesting that most of the LsMVP protein was located in the genital segment, containing developing oocytes and cement glands. A separate analysis of the cement gland and oocytes revealed a high enrichment of LsMVP in the cement gland, while no protein was detected in the oocytes (Fig. 4e).

Finally, to investigate whether LsMVP assembles into vaults, differential centrifugation of salmon louse tissue lysate was performed. Intact vaults are known to pellet at 100,000 × g, also known as the microsomal pellet^1^. Western blot analysis detected LsMVP in the microsomal pellet (100P) compared to the supernatant (100S), which suggests that vaults most likely are present in the salmon louse (Fig. 4f). Some lower bands were detected in the 100P sample, which are likely some degradation products.

### LsMVP is a component of the egg string forming cement material

**Figure 5:**
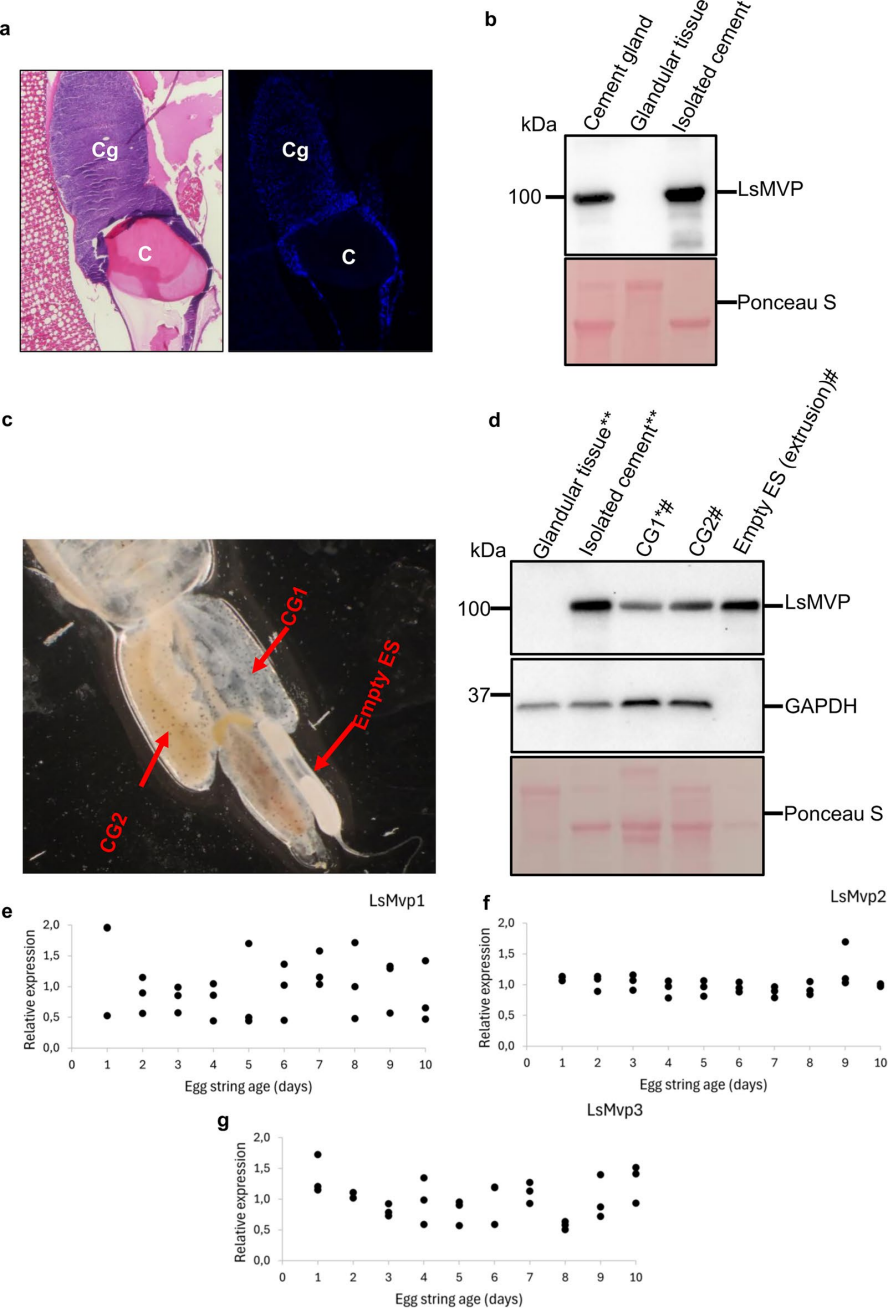
Major vault protein is secreted into the cement. (**a**) HE- and DAPI staining of the cement gland (Cg) and cement (C). No nuclear staining was detected in the cement (C), indicating that it is an extracellular material. (**b**) A western blot analysis showing that LsMVP is present in cement and not in glandular tissue. (**c**) Image of an adult female salmon louse with ovary disease. (**d**) A cropped western blot showing analysis of samples from an adult female salmon louse with ovary disease. LsMVP was detected in the empty egg string that consists of cement and without any embryos. (**e–g**) Relative gene expression analysis (E^−ΔΔCt^) of *LsMvp1,* *LsMvp2* and *LsMvp3* in adult female salmon lice during an egg string cycle (N = 3). No apparent change was observed for either of the transcripts. *CG* cement gland, *ES* Egg string, *Gland where empty egg string was extruded from, ^#^from the same individual, **from the same cement gland. The original blot images are shown in Supplementary Fig. S8.

Two cement glands are found in the genital segment of the female salmon louse. As oocytes are fertilized and extruded, the glands secrete a substance called cement that covers the eggs and serves as a protective layer of the egg string. To understand the nature of the cement secretions, a section of the cement gland was stained with the nuclear monitoring stain DAPI. Imaging showed nuclei staining only in the glandular tissue and not in the cement (Fig. 5a), indicating that the cement is an extracellular material. Western blot analysis showed LsMVP to be present in the isolated cement, while no protein was detected in the glandular tissue (Fig. 5b). The presence of LsMVP in the cement was also confirmed by mass spectrometry analysis of the putative LsMVP band cut out from an SDS PAGE gel that identified peptides of LsMVP1 and LsMVP2 (see Supplementary File S1).

It has been observed that in some cases the adult female salmon louse does not produce any oocytes (usually in one side) due to a disease condition affecting the ovary or the oviduct. In these cases, the female salmon louse is still producing an egg string sac consisting of the cement material, but without any fertilized embryos (Fig. 5c). Analysis of extracts from such an empty egg string sac revealed the presence of high amounts of LsMVP (Fig. 5d), suggesting that LsMVP is an architectural component of the salmon louse egg string that is incorporated during its formation. The presence and enrichment of LsMVP in the cement was surprising and we wondered whether it was a distinguishing characteristic of the salmon louse or an evolutionary conserved trait of the *Caligidae* family of sea lice. Therefore, we investigated the presence of MVP in *Caligus* *elongatus,* a sea louse from a different but related genus. Similar to the salmon louse, *C.* *elongatus* MVP was enriched in the female genital segment while the protein abundance in males was barely detectable in comparison (see Supplementary Fig. S4).

We then investigated if the level of *LsMvp* transcripts changed during an egg producing cycle as the cement gland is emptied upon egg string extrusion and new cement is produced for the next egg string production. Relative gene expression of *LsMvp* in whole female salmon lice at different days of egg string maturation was measured. No apparent change in relative gene expression was observed during the 10 days of egg string maturation for any of the three transcripts (LsMvp1-3) (Fig. 5e–g). Finally, RNAi experiments were performed to assess whether a knockdown of *LsMvps* affected the production of the cement and egg strings. No phenotype was observed 34 (experiment 1) or 41 (experiment 2) days after injection with dsRNA (see Supplementary Figs. S5 and S6), and the adult female salmon lice were producing normal egg strings with successful hatching. A knockdown efficiency of 80% (*LsMvp1*) and 68% (*LsMvp2/3*) for experiment 1 and 60% (*LsMvp1*) and 53% (*LsMvp2/3*) for experiment 2 was measured by relative gene expression analysis. The number of recovered salmon lice was not significantly different in the knock-down groups from the control groups (see Supplementary Table S3).

## Discussion and conclusion

In the present study, we have shown that MVP is present in the majority of ecdysozoan species, with three full-length MVP paralogues found in the Atlantic salmon louse. Gene and protein expression analyses showed that LsMVP is differently expressed in various tissues, with a high enrichment of the protein in the extracellular cement material which is known to form the outer shell of the female salmon louse egg strings.

MVP is the main component of the vault ribonucleoprotein complex and is present in a large diversity of eukaryotic species. Most eukaryotic species have been shown to have one single MVP paralogue, except for *Dictyostelium* which has at least two paralogues^1^. In this study, we identified three paralogues of MVP in the Atlantic salmon louse. Two of the genes (LsMvp2 and LsMvp3) show a high sequence identity, indicating a recent gene duplication event. It was previously reported that no MVP orthologues could be found in the Arthropoda^25^. However, our comprehensive database search for MVP in ecdysozoan species indicates the probable presence of MVP orthologues across most ecdysozoan lineages. This finding suggests a higher number of known ancestral and independent gene loss events in this group. We did not see any obvious correspondence between habitat or lifestyle and gene loss of Mvp. Further, strictly terrestrial clades such as Onychophora, Arachnidae (minus Acari), or basal insects did not show gene loss. In-depth comparative analyses of sister groups with and without orthologues such as Malacostraca and Maxillopoda, or embedded groups with gene loss, such as the Acari within the other Celicerata may help to elucidate MVP and vault function in Metazoa. In this context, our discovery of MVP-like sequences in two marine nematodes with plausible placement in the metazoan tree is noteworthy. However, genomic data from marine nematodes is still extremely scarce and the coverage of most clades should be improved.

Furthermore, we investigated the localization and expression of both LsMVP transcript and protein. *LsMvp* transcripts were shown to be highly expressed in the intestine and subcuticular tissue, while the protein was shown to be mostly present in the cement. Expression of *LsMvp* in the intestine was not unexpected, as expression of MVP has previously been observed in the epithelial cells of the intestine in other species such as humans^10^, fish^11^, and rats^53^, suggesting that there is a common functional role of MVP in this tissue.

Little is known about the subcuticular tissue, although a microarray assay of five different tissues in the salmon louse showed that genes involved in fatty acid degradation, fatty acid elongation, degradation of amino acids, synthesis of Acetyl CoA and the citrate cycle were upregulated in the subcuticular tissue^54^. The subcuticular tissue is also, among others, the main production site for the yolk-related proteins LsVit1, LsVit2, and LsYAP^50,55^. The yolk proteins are known to be secreted into the hemolymph in the subcuticular tissue and transported to the developing oocytes in the genital segment^55^. One explanation for the low transcript levels in the cement gland compared to the subcuticular tissue and the high protein abundance in the cement compared to the subcuticular tissue could be due to the transport of LsMvp from the subcuticular tissue to the cement gland, although the mRNA and protein expression levels are not always correlated. Attempts to extract hemolymph to test this hypothesis have, however, not been successful due to technical difficulties. When studying the localization of LsMVP mRNA and protein in the subcuticular tissue, we did notice that the mRNA and protein seem to be localized differently. LsMVP protein seems to be localized to some type of fibrous/connective tissue, while the transcript seems to be located in nearby cells, suggesting that LsMVP might be secreted. However, this needs to be further investigated.

LsMVP protein is predominantly present in the cement of the female salmon louse. However, it remains unclear whether LsMVP is present in the cement as vaults or single MVP, or what functional role it has. Working with cement has been challenging due to its nature, and investigation into the specific molecular function of LsMVP was hindered by the cement insolubility in non-denaturing buffers. We presume that the cement, and hence LsMVP, is progressively secreted by the cement gland’s secretory cells into the gland’s central canal in synchrony with salmon louse reproductive cycles before its release during egg string extrusion and that is why the amount of LsMVP in the cement gland is higher compared to other salmon louse tissues. RNA interference (RNAi) experiments have previously been performed on some of the main components of cement, giving clear phenotypes with female salmon lice having either abnormal egg strings or no egg strings at all^28^. Similar RNAi experiments were performed on *LsMvp*, but no divergent phenotype from the control salmon lice was observed. Gene expression analysis showed the highest knockdown efficiency to be 80% for *LsMvp1* and 68% for *LsMvp2/3*, which might not have been sufficient to give a phenotype that we could identify. This finding is similar to those of other studies in which MVP-deficient mice did not show any obvious phenotypic abnormalities^18,56^.

Most studies have previously shown that MVP or vaults are mainly intracellular particles that localize to the cytoplasm and to a lesser extent in the nucleus^21^. However, there are a few studies suggesting that MVP can be a secretory protein. Lee et al.^57^ reported that MVP was localized on the cell surface of hepatocellular carcinoma (HCC) cell lines, but not on normal hepatocytes. They also showed that cell surface MVP was induced by environmental stress and to be regulated by ERK and mTOR signaling pathways. Another study by Teng et al.^58^ showed that MVP was associated with miR-193a in exosomes in colon cancer cells. Reviewing mass spectrometry (MS) analyses from previous studies show the presence of MVP-like proteins in cement secretions from barnacles, which are sessile marine crustacea that secrete cement for attachment to solid substrates under water^59–62^. While the cement in sea lice and barnacles are not directly analogous substances, the presence of MVP in both secretions strongly suggests that MVP homologues are secretory and architectural components of cement in Crustacea. Additionally, MVP is one of the frequently identified proteins in the ExoCarta exosome database^63^ and is also one of the protein components identified by MS analysis of senescent secretory phenotype (SASP) secretome, stem cells-derived exosomes, extracellular vesicles produced by activated mouse hepatic stellate cells and non-vesicular extracellular matter of colon cancer and glioblastoma cells^64–67^.

In conclusion, these findings indicate a novel function of MVP as a secretory cement protein in the female salmon louse. The elucidation of LsMVP as a cement-related protein extends our knowledge of the biomolecular composition and chemical properties of the cement and thereby the egg strings which play a fundamental role as protective coats during embryogenesis in the salmon louse and similar organisms.

### Supplementary Information

Supplementary Information 1.

Supplementary Information 2.

Supplementary Information 3.

## Data Availability

The sequence data presented in this study have been deposited in GenBank repository (www.ncbi.nlm.nih.gov/genbank/) with the following accession numbers: PP449126, PP449127, PP449128, and PP449129. The mass spectrometry proteomics data have been deposited to the ProteomeXchange Consortium via the PRIDE^68^ partner repository with the dataset identifier PXD050812 and 10.6019/PXD050812.
