# Supplementary material for: Major vault protein is part of an extracellular cement material in the Atlantic salmon louse (Lepeophtheirus salmonis)
Source: Sci Rep. 2024 Jul 2;14:15240. doi: 10.1038/s41598-024-65683-0 (PMC11219742; doi:10.1038/s41598-024-65683-0)
Supplement: Supplementary file 3 — Supplementary Information 3. [file 41598_2024_65683_MOESM3_ESM.pdf]

Major vault protein is part of an extracellular cement material in the Atlantic salmon louse (*Lepeophtheirus salmonis*)

Supplementary Information

Author information:

Malene Skuseth Slinning[1#], Thaddaeus Mutugi Nthiga[1#], Christiane Eichner[1], Syeda Khadija[3], Leonard H. Rome[3], Frank Nilsen[1], Michael Dondrup[2*]

[1] Sea Lice Research Centre (SLRC), Department of Biological Sciences, University of Bergen, Pb. 7803, 5020, Bergen, Norway

[2] SLRC, Computational Biology Unit (CBU), Department of Informatics, University of Bergen, Pb. 7803, 5020, Bergen, Norway

[3] Department of Biological Chemistry, David Geffen School of Medicine and the California NanoSystems Institute, University of California Los Angeles, Los Angeles, CA 90095, USA.

#Equal contribution

Corresponding author:

Correspondence to Michael Dondrup (Michael.dondrup@uib.no)

Supplementary Data

Supplementary File S1: Peptides identified by Proteome Discoverer. A band around 100 kDa from an SDS-gel was excised for protein identification in the cement by mass spectrometry. Peptides matching LsMvp1 and LsMvp2 were identified.

Supplementary File S2: FASTA file containing the multiple sequence alignment of MVP orthologues that were used to create the maximum-likelihood phylogenetic tree in Figure 2a.

Supplementary Figures

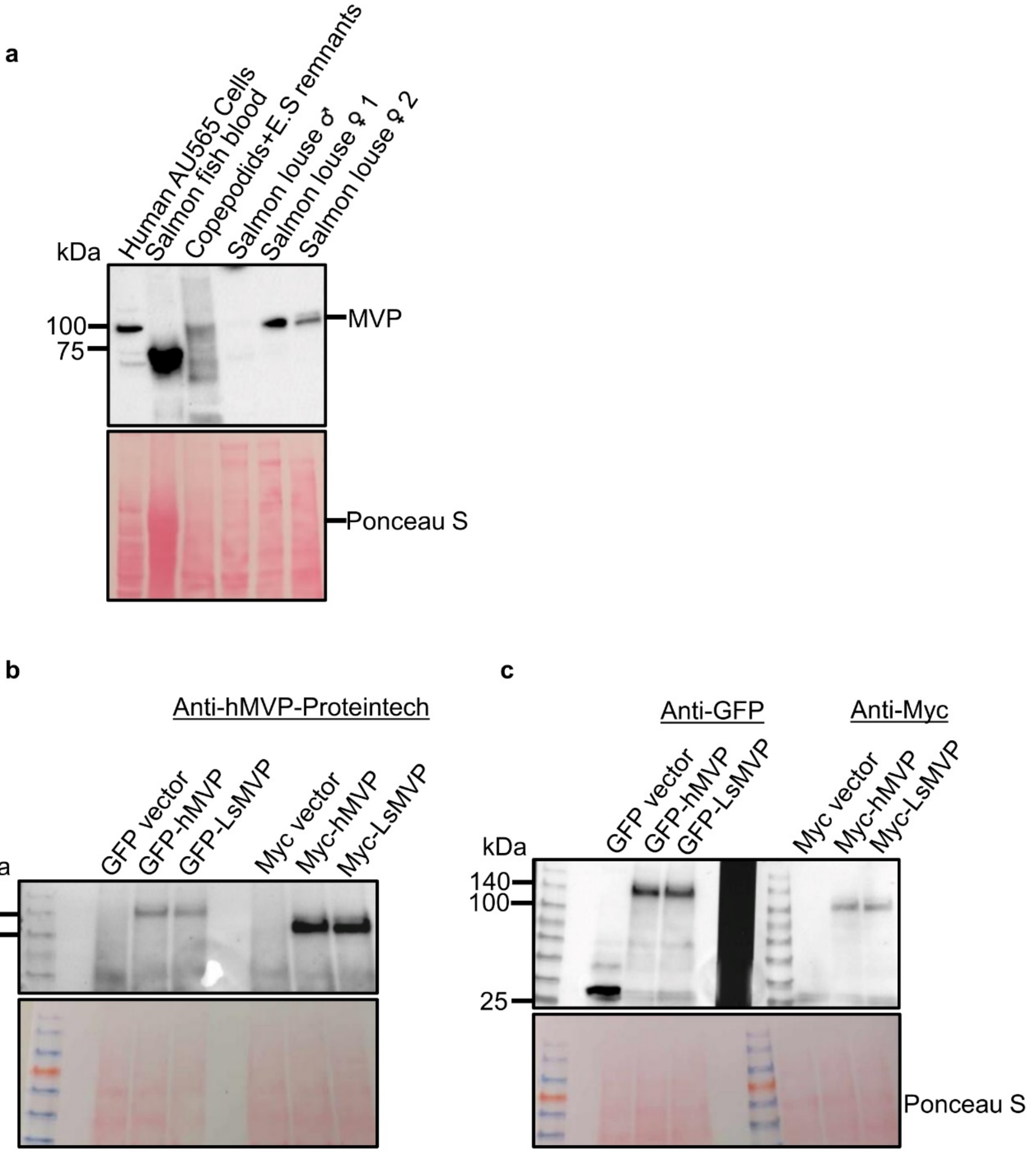

Supplementary Figure S1: Anti-human MVP antibody recognizes salmon louse MVP (LsMVP). **a)** Immunoblot analysis of extracts from human AU565 cells and salmon louse using anti-human MVP antibody. **b, c)** Immunoblot analyses of human and salmon louse (LsMVP2) *in vitro*-translated GFP- and Myc tagged MVP probed with anti-human MVP **(b)**, anti-GFP and anti-Myc **(c)** antibodies. Abbreviations: E.S-egg string, hMVP- human MVP, LsMVP-salmon louse MVP. Original blot images are shown in Supplementary Figure S8.

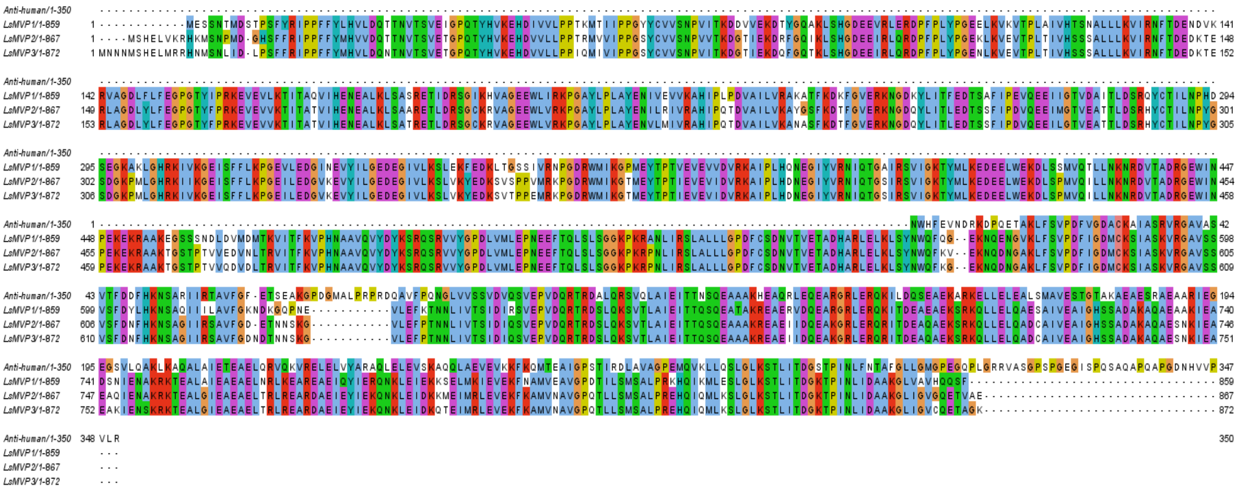

Supplementary Figure S2: Multiple sequence alignment between LsMVP1-3 proteins and the epitope sequence recognized by the anti-human MVP antibody (residues 544-893). LsMVP1 shares 49% identity with the epitope sequence, while LsMVP2 and LsMVP3 share 54% identity with the epitope sequence.

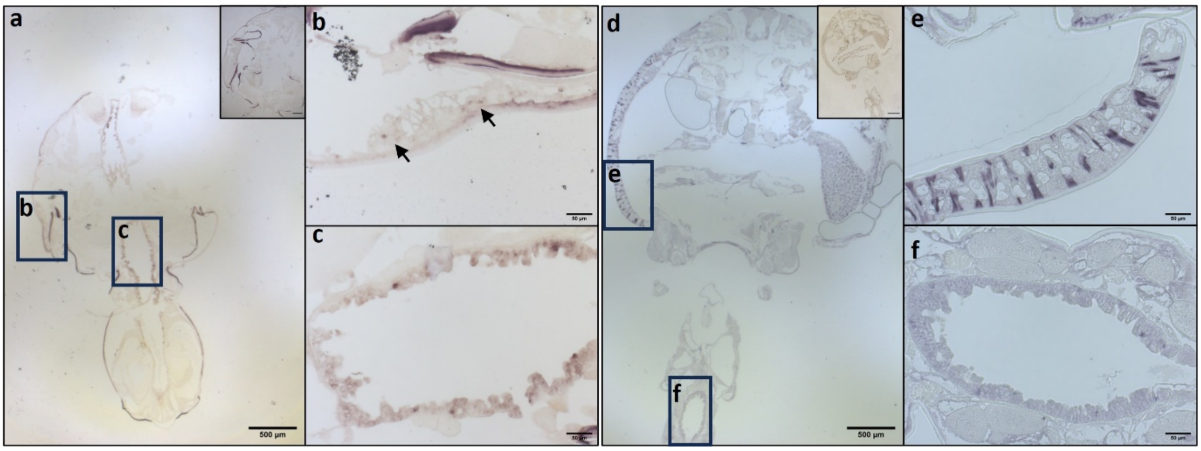

Supplementary Figure S3: mRNA and protein localization of LsMVP in adult male salmon louse (*L. Salmonis*). a) *In situ* hybridization was performed on adult male salmon louse sections to determine localization of *LsMvp* mRNA expression. Ls*Mvp* mRNA expression was localized to **b)** the subcuticular tissue and **c)** intestine. **d)** Immunohistochemistry was performed to determine the localization of LsMVP protein. LsMVP protein was localized to **e)** the subcuticular tissue and **f)** intestine. Scale bar = 500 µm (**a**, **d**) and 50 µm (**b**, **c**, **e**, **f**). Controls shown upper right in boxed images (scale bar = 200 µm (**a**) and 500 µm (**d**)).

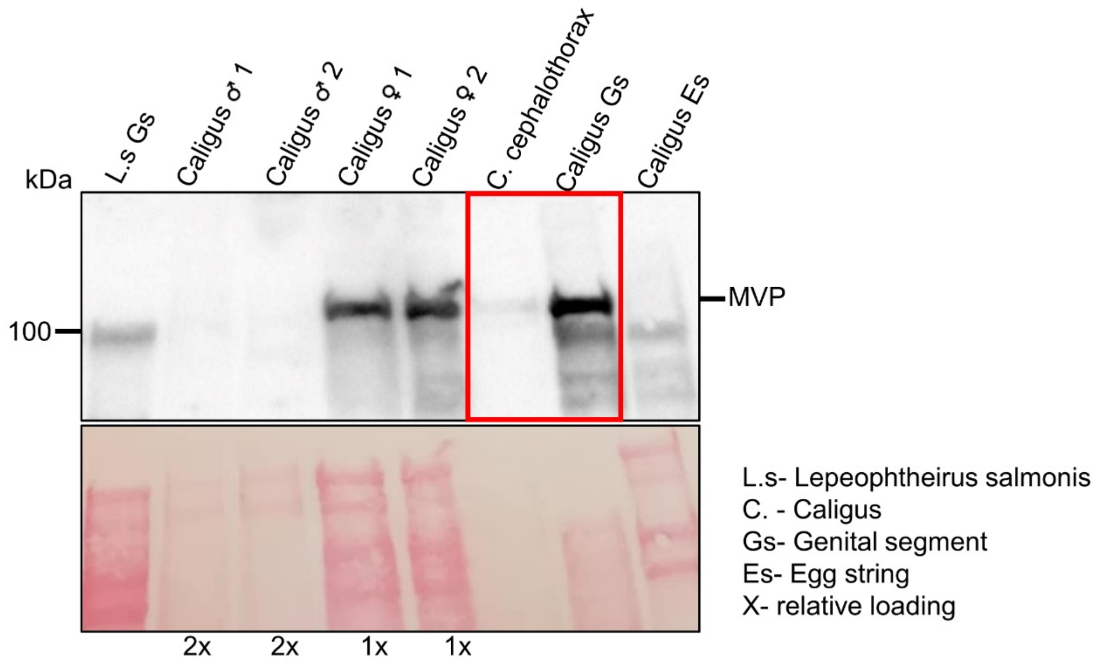

Supplementary Figure S4: Western blot analysis showing MVP is enriched in the genital segment of female *Caligus elongatus*. The original blot image is shown in Supplementary Figure S8.

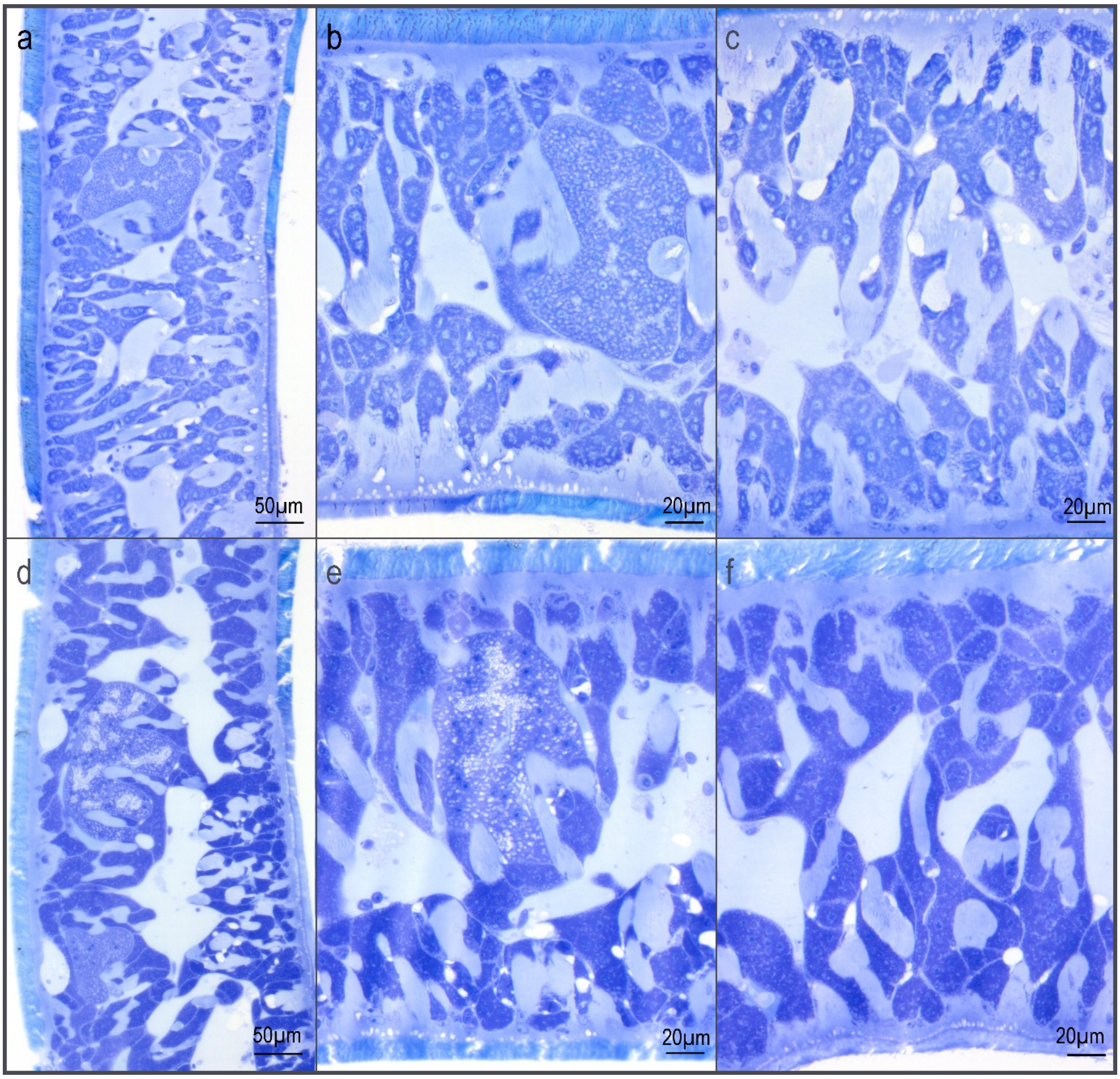

Supplementary Figure S5: Knockdown of *LsMvp*1-3 by RNA interference. Histology images of subcuticular tissues of the cephalothorax of an adult female salmon louse (*L. salmonis*) in **a-c)** the control group and **d-f)** the *LsMvp* knockdown group. Scale bar = 50 µm (**a**, **d**) and 20 µm (**b**, **c**, **e**, **f**).

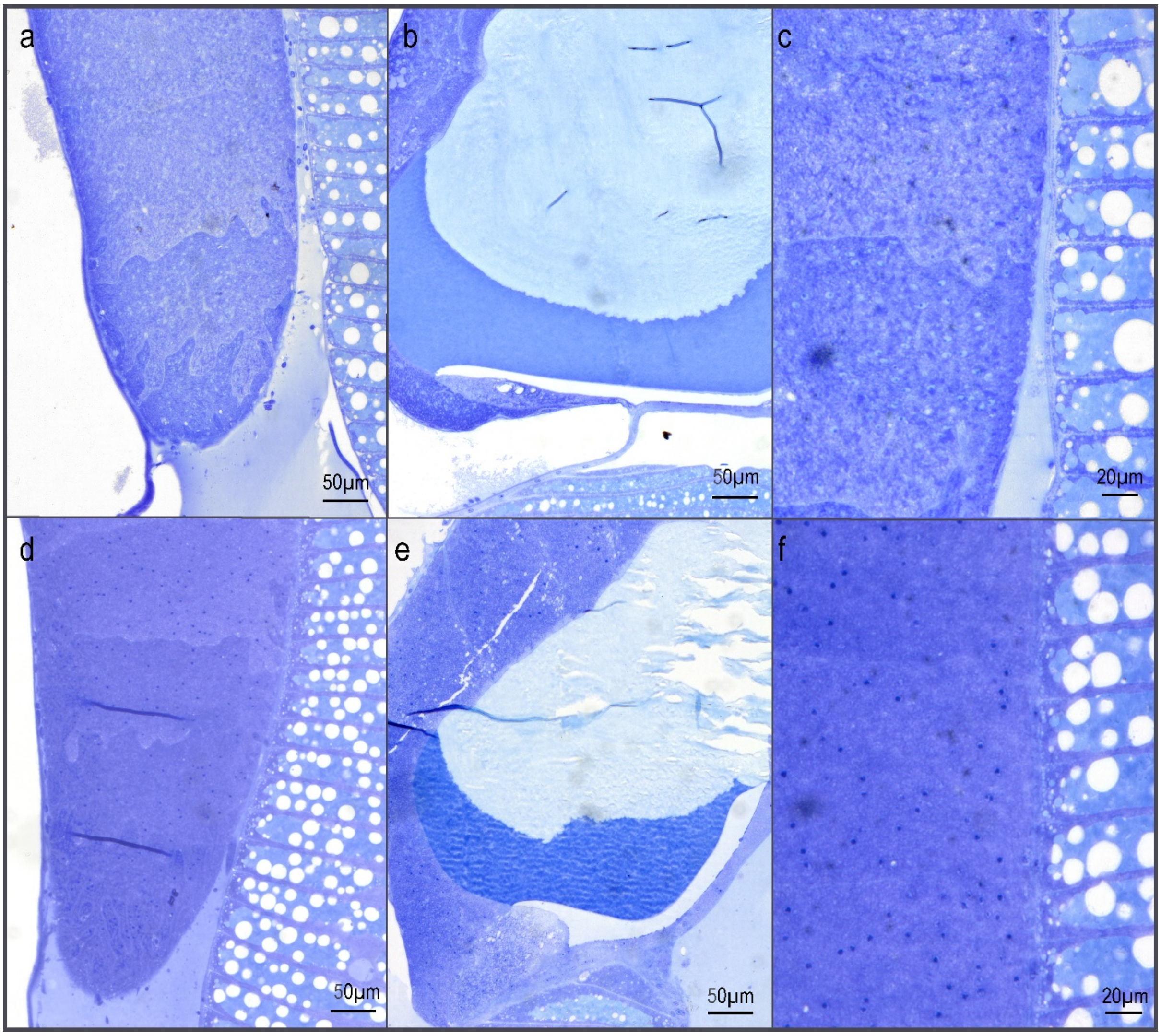

Supplementary Figure S6: Knockdown of *LsMvp*1-3 by RNA interference. Histology images of the cement gland and cement of an adult female salmon louse (*L. salmonis*) in **a-c)** the control group and **d-f)** the *LsMvp* knockdown group. Scale bar = 50 µm (**a**, **b**, **d**, **e**) and 20 µm (**c**, **f**).

```
LsMvp1      ------------------------------------------------------------    0
LsMvp2      agtctattgattatttataataggctgcagagttttcaagtcaacccatttcattgccta    60
LsMvp3      -----------------------------------------------attttcattccta    13


LsMvp1      ---------------------------------------------attcctaagttaat-    14
LsMvp2      tttgtacttatttcgactc---------acatcattcttaatagagttggtgaactaata    111
LsMvp3      tttgtacatactttgagtcgcatcattcttaatagtctatagagagttggtgaactgata    73
                                                          **  * *  * **

LsMvp1      --------------ttttcaaggagacggaatcaacattttgttaattagagaacatctc    60
LsMvp2      ttgtgataggatacaattcagctccaggaactcatcaattcttaagctacataacattgc    171
LsMvp3      ttgtgatagaattcacttcaactccaggaacgcatcaattctaaagatacataacattgt    133
                            ****     * * *  ** ** **    *  ** * *****

LsMvp1      ctctga----tccccatatttaataatacatcggaaaaatggaaagttcgaacactatgg    116
LsMvp2      tagta-------ataatatgtcccatgagcttgtgaaaagacataaaatgtcaaatccaa    224
LsMvp3      tagtaatgaataataatatgtctcatgagcttatgagaagacataatatgtcaaatctaa    193
               *           **** *   *  *  *    * **   * *    *   * *

LsMvp1      attcaactccctcattctataggattccaccatttttttatttgcacgttctggatcaaa    176
LsMvp2      tggatggacattcattctttcgaatccctcctttttttctacatgcacgttgtggatcaaa    284
LsMvp3      ttgatttaccttcattctttcgaatccctcctttttttctacatgcacgtcctggatcaaa    253
                    *  ******* * * ** ** ** ***** **  *******  *********

LsMvp1      ctaccaatgtcacatcagtagaaataggcccccaaacatatcatgtaaaggaacatgaca    236
LsMvp2      ccacaaatgttacatccgttgaaactgggccacaaacctaccatgtgaaggaacacgacg    344
LsMvp3      acactaacgttacatccgttgaaactgggccacaaacctaccatgtgaaggaacatgacg    313
              ** ** ** ***** ** ****  ** ** ***** ** ***** ******** ***

LsMvp1      tagttgttcttccgcctacaaaaatgacaattattcctcctggttattattgtgtcgtat    296
LsMvp2      ttgtcctcctccctcccacccgaatggtcgtaatacctccaggatcttattgtgttgtat    404
LsMvp3      ttgtcctcctccctcccattcaaatgatagtaatccctccaggatcttattgcgttgtat    373
            * **  * ** ** ** *    ****    * ** ***** ** * ****** ** ****

LsMvp1      cgaatccagttattacaaaggatgatgttgttgaaaaggatacatacggacaagctaaat    356
LsMvp2      caaaccctgttgtaacaaaagatggtactattgagaaagatcgctttggccaaattaagt    464
LsMvp3      caaaccctgttataacaaaagatggtactattgagaaagatcagttcggccaaactaagt    433
            * ** ** *** * ***** **** *  * **** ** ***   *  ** ***  *** *

LsMvp1      tgtcacatggagacgaagaagtgcgattggagagagatccgtttccattatatccaggag    416
LsMvp2      tatctcatggagatgaagaaattcgattacaaagagatccttttccattatatccaggag    524
LsMvp3      tatctcatggagacgaagaaattcgattacaaagagacccttttccattatatccaggag    493
            * ** ******** ****** * *****  * ***** ** *******************

LsMvp1      aagagttaaaggtcaaggtcacaccattggcaatcgttcatacctcaaatgcccttttat    476
LsMvp2      agaaattgaaagttgaagtgacgcctttaacaatcgtacattcatcaagtgcacttcttt    584
LsMvp3      agaatttgaaagttgaagtaacgcctttaacaatcgtacattcatcaagtgcacttcttt    553
            *  * ** ** **  * ** ** ** **  ******* *** * **** *** *** * *

LsMvp1      taaaagttataagaaatttcacggacgaaaatgatgttaaacgtgtggctggagatttat    536
LsMvp2      taaaagttatcagaaattttactgatgaagacaaaactgagcgtcttgctggagatttat    644
LsMvp3      taaaagttatcagaaattttactgatgaagacaaaactgagcgtcttgctggagatttat    613
            ********** ******** ** ** *** *  *   * * *** * *************

LsMvp1      ttttgtttgagggacctggaacttatattccaaggaaagaggtcgaggttcttaaaacca    596
LsMvp2      acttgttcgaaggtccaggaacttattttcctagaaaagaggttgaagttgttaaaacta    704
LsMvp3      acttgttcgaaggtccaggaacttattttcctagaaaagaggtggaagttgttaaaacta    673
              ***** ** ** ** ********* **** ** ******** ** *** ******* *

LsMvp1      ttacagcacaagttatccatgaaaatgaagcccttaaattaagtgcaagtcgtgaaacca    656
LsMvp2      ttactgctacagtcatacatgagaatgaagctttaaaactgagtgctgctcgtgaaactt    764
LsMvp3      ttactgctacagtcatacatgagaatgaagctttaaaacttagtgctactcgagaaactt    733
            **** **   *** ** ***** ********  * *** * *****   *** *****

LsMvp1      tagatcgatccggaattaaacacgttgcaggtgaagaatggctcataagaaagcctggag    716
LsMvp2      tagacagatctggttgtaaaagagtggcaggggaggaatggttagtaagaaaacctggag    824
LsMvp3      tggacagatctggttgtaaaagagtggcaggggaggaatggttggttagaaaacctggag    793
            * **  **** **   ****   ** ***** ** ****** *  * ***** *******

LsMvp1      cttatttgcctttggcttatgaaaatattgtggaagttgttaaggcacatataccccttc    776
LsMvp2      cttatctccctctcgcgtatgaaaatattcttaggattgttagagctcatataccacaaa    884
LsMvp3      cttatctccctctcgcgtatgaaaatgttcttatgattgttagagctcacataccacaaa    853
            ***** * *** * ** ********* ** *     ******  ** ** ***** *

LsMvp1      cagatgtcgcaattttggtaagagcaaaggcaactttcaaggacaagtttggtgttgaga    836
LsMvp2      cagatgttgctattttagtcaaggcctatggatcctttaaagatacatttggagttgaac    944
LsMvp3      cagatgttgctattttagtcaaggctaatgcatcctttaaagatacatttggagttgagc    913
            ******* ** ***** ** *  **  * * * * ** ** ** *  ***** *****

LsMvp1      gaaaaaatggagacaaatacttgattacctttgaagatacatcagcattcattcctgagg    896
LsMvp2      ggaagaacggagaccaatatttaataacccttgaagatacatcatccttcatacccgatg    1004
LsMvp3      ggaagaacggagaccaatatttaataaccctggaagatacatcatccttcatacccgatg    973
            * ** ** ****** **** ** ** *** * ************ * ***** ** ** *

LsMvp1      tacaagaagaaataattggcacagtggatgctatcacactggatagtcgacagtactgca    956
LsMvp2      ttcaagaagaaattatgggtactgtcgaagcaactacacttgatagtagacattattgta    1064
LsMvp3      ttcaagaagaaattttgggtactgttgaagccactacacttgatagtagacattattgta    1033
```

```
               * ***********  * ** ** ** ** ** *  ***** ****** **** ** ** *

LsMvp1         caatattaaacccacatgattcagaagggaaggcaaagcttggacatagaaaaattgtaa    1016
LsMvp2         ccattcttaatccatatggttcagatgggaaacccatgcttggacatcggaaaattatca    1124
LsMvp3         ccattcttaacccctatggctcagatgggaaacccatgcttggtcatcgaaaaattgtca    1093
               * **  * ** **  ***  ***** *****  * * ****** *** * ****** * *

LsMvp1         aaggagaaatttcatttttcctgaaaccaggagaagtactcgaggacggtatcaatgagg    1076
LsMvp2         aaggagaaatttcatttttttcttaagccaggagaaattcttgaggatggtgtgaaggaag    1184
LsMvp3         aaggggaaatctcatttttttcttaagccaggagaaattcttgaggatggtgtgaaggaag    1153
               **** ***** ******** ** ** ********* * ** ***** *** * ** ** *

LsMvp1         tatacattcttggagaagatgaaggtatcgttctaaagtctttggagaagtttgaagata    1136
LsMvp2         tttacattcttggtgaggatgagggtattgtcttaaagtccttggttaagtacgaggata    1244
LsMvp3         tttacattcttggtgaggatgagggtattgtcttaaagtccctggttaagtacgaggaca    1213
               * *********** ** ***** ***** **  *******  ***  ****  ** ** *

LsMvp1         aattaacaggctcttcaatcgtcagaaatcctggagatagatggatgattaaaggaccaa    1196
LsMvp2         agtcagtctctcccccagtaatgcgaaaacctggagatagatggatgataaaaggaacta    1304
LsMvp3         agtcagtgactcccccagaaatgcgaaaacctggagacagatggatgataaaaggaacta    1273
               * * *       *  **    *  **** ******** *********** ****** * *

LsMvp1         tggagtatacaccgacagttgaagtagaggttgttgacgtacgaaaagcaatacccttac    1256
LsMvp2         tggaatatactcctacgattgaggtagaagtaatagacgtaagaaaagcaatacctctgc    1364
LsMvp3         tggaatatactcctactattgaggtagaagtcatagacgtaagaaaagcaatacccttgc    1333
               **** ***** ** **  **** ***** **  * ****** *************  * *

LsMvp1         atcaaaacgaaggaatttacgttagaaatattcaaactggtgcaatcagatccgttatag    1316
LsMvp2         atgacaatgaaggaatttacgttcgaaacattcagactggttctataagatcagttattg    1424
LsMvp3         atgacaatgaaggaatttacgttcgaaacattcagactggttctataaggtcagttattg    1393
               ** * ** *************** **** ***** ****** * ** ** ** ***** *

LsMvp1         gaaaaacgtacatgctcaaagaggatgaagagctttgggaaaaagatttgtcatctatgg    1376
LsMvp2         gtaaaacttatatgcttaaagaagatgaagaactctgggagaaggatctatcacccatgg    1484
LsMvp3         gtaaaacttatatgcttaaagaagatgaagaactctgggaaaaggatctatcacccatgg    1453
               * ***** ** ***** ***** ******** ** ***** ** *** * *** * ****

LsMvp1         ttcaaactttattaaataaaaatagggatgtcactgctgatagaggcgaatggattaatc    1436
LsMvp2         tacaaattttgttgaataaaaatcgggatgttacagcagatagaggtgaatggattaatc    1544
LsMvp3         tacaaattctgttgaataaaaatcgggatgttacagcagatagaggggaatggattaatc    1513
               * **** * * ** ********* ******* ** ** ******** *************

LsMvp1         cagagaaggagaaaagagctgcaaaggagggttccagtagcaatgatctcgatgtcatgg    1496
LsMvp2         ctgaaaaagaaaaaagagctgctaaaactggctcaaccccaacagtagttgaagatgtca    1604
LsMvp3         ctgaaaaagaaaaaagagctgctaaaactggctcgaccccaacagtagttcaagatgtcg    1573
               * ** ** ** *********** **    ** ** *     *  *   *  * *   *

LsMvp1         atatgacaaaagttataacatttaaagttcctcataatgccgctgtccaagtctatgatt    1556
LsMvp2         atcttactcgagtaataacctttaaagttccacacaatgcggcagttcaagtatatgact    1664
LsMvp3         atcttactcgagtaataacctttaaagttccacacaatgcggcagttcaagtatatgact    1633
               ** * **   *** ***** *********** ** ***** ** ** ***** ***** *

LsMvp1         ataagagtcgacagtctagagttgtttatggacctgatctcgtaatgttggagcccaacg    1616
LsMvp2         ataagagccgtcaatcaagagtagtgtatggtcccgatttagttatgcttgagcctaatg    1724
LsMvp3         ataagagccgtcaatcaagagtagtgtatggtcccgatttagttatgcttgaacctaatg    1693
               ******* ** ** ** ***** ** ***** ** *** * ** *** * ** ** ** *

LsMvp1         aggagtttacacaactttctcttagtggaggaaaacctaaaagggctaatttaattcgtt    1676
LsMvp2         aggaatttactcagttatctcttagtggaggaaaaccaaaacggcccaatcttatccgtt    1784
LsMvp3         aggaatttactcagctatctcttagtggaggaaaaccaaaacgacccaatcttatccgtt    1753
               **** ***** **  * ******************** *** *  * *** * ** ****

LsMvp1         ccttggccttgctcctaggtccagatttttgttcagacaatgtaaccgttgaaacggcag    1736
LsMvp2         cacttgctttactgttaggaccagatttttgttctgataatgtcactgttgaaactgccg    1844
LsMvp3         cacttgctttactgttagggccagatttttgttctgataatgtcactgttgaaactgctg    1813
               *  * ** ** **  **** ************** ** ***** ** ******** ** *

LsMvp1         atcatgcaagattagaattgaaactctcttataactggcaatttcaaggagaaaagaatc    1796
LsMvp2         atcatgctagactagagttaaagctttcctacaattggcaatttaaagttgaaaagaatc    1904
LsMvp3         atcatgccagactagagttaaagctttcctacaattggcaatttaaaggtgagaagaatc    1873
               ******* *** **** ** ** ** ** ** ** ** ********* ***  ** *******

LsMvp1         aagagaacggagttaaattgttcagtgttcctgattttattggagatatgtgcaaatcca    1856
LsMvp2         aagataatggagcaaagttatttagcgtccctgactttatcggagatatgtgcaaatcca    1964
LsMvp3         aagataatggagcaaagttatttagcgtccctgactttattggagatatgtgcaaatcca    1933
               **** ** ****  ** ** ** ** ** ***** ***** *******************

LsMvp1         ttgcgtcgaaagtaagagggggccgtttcatccgtatcatttgattatctccataaaaact    1916
LsMvp2         ttgcttcaaaagtgagaggagcggtctcctcagtttctttcgataattttcataaaaatt    2024
LsMvp3         ttgcttcaaaagtgagaggagcggtttcctcagtttctttcgataacttccataagaatt    1993
               **** ** ***** ***** ** ** ** ** ** ** ** *** *  * ***** ** *

LsMvp1         ctgcacaaataattattttagctgtatttgggaagaatgataaaggtcaaccaaatgagg    1976
LsMvp2         ctgcagggataattcgatcggccgtatttggagac---gagacaaataattcgaagggcg    2081
LsMvp3         ctgcggggataattcgctctgctgtatttggagacaacgatacaaataattcaaagggag    2053
               ****    ******   *  ** ********  *    ** * *  * *  * ** *  *

LsMvp1         tcctggaatttaagacaaacaaccttatagttacctcaattgatattagaagtgtcgagc    2036
```

```
LsMvp2      tcttggaattccccaccaataaccttatagttacatccatcgatatccaaagtgttgaac    2141
LsMvp3      tcctggaattccccaccaataaccttatagttacatccatcgatattcaaagtgttgagc    2113
            ** *******    ** ** ************* ** ** *****   ****** ** *

LsMvp1      ctgtggatcagcgaacgcgtgattctcttcaaaagtctgtaaccctagccatagaaataa    2096
LsMvp2      ctgttgatcaacgaacacgagattcactccaaaagtctgtaaccctcgccatagaaatta    2201
LsMvp3      ctgttgatcaaagaacacgagattcactacaaaagtctgtaacccttgccatagaaatta    2173
            **** *****  **** ** ***** ** **************** *********** *

LsMvp1      ctactcagtcacaggaggctacagcaaagagagaagcagaaagggtggaccaagaagcaa    2156
LsMvp2      caactcaatcccaagaagctgcagctaaaagagaagcagaaataattgaccaagaagcca    2261
LsMvp3      caactcaatcccaagaagctgcagctaaaagggaagcagaaataattgaccaagaagcta    2233
            * ***** ** ** ** *** **** ** ** **********   * *********** *

LsMvp1      gaggcagactagagcgacaaaaaattacggacgaggcagaagcagaaaaatcgcgcaaac    2216
LsMvp2      aaggtcgattagaaagacaaagaatcacagatgaagcacaggcagaaaaatctcgaaaac    2321
LsMvp3      aaggtcgattggaaagacaaagaatcacagatgaagcacaggcagaaaaatctcgtaaac    2293
             ***   ** * **  ****** *** ** ** ** *** * *********** ** ****

LsMvp1      aacttttagaattacaagcagaaagtgctatagtggaagcaatcggtcattcaagtgcag    2276
LsMvp2      aacttttggaattacaggctgattgtgctattgttgaggccattggtcactccagcgcag    2381
LsMvp3      aacttttggaattgcaggctgattgtgctattgttgaggccattggtcactccagcgcag    2353
            ******* ***** ** ** **  ******* ** ** ** ** ***** ** ** ****

LsMvp1      atgctaaagctcaagcagaagcagccaaaattgaagcagactcaaatatcgaaaatgcaa    2336
LsMvp2      atgctaaagctcaagctgaatctaacaaaattgaagctgaggctcaaattgaaaatgcaa    2441
LsMvp3      atgctaaagctcaagctgaatcgaacaaaattgaagctgaggctaaaattgaaaattcaa    2413
            *************** *** *   *********** **  *  * ** ****** ***

LsMvp1      aacgtaagactgaagccttggccattgaggcagaagcagaactgaatcgactcaaagagg    2396
LsMvp2      aaagaaaaactgaagctctaggtattgaagctgaagcagaacttactcgccttagagaag    2501
LsMvp3      aaagaaagactgaagctctaggtattgaagctgaagcagagcttactcgccttagagaag    2473
            ** * ** ********  * *  ***** ** ******** ** * *** ** * *** *

LsMvp1      ccagagaggcagagatccaatatattgaaagacagaataaactagaaatagagaaaaaaa    2456
LsMvp2      ctcgtgatgccgaaattgagtatattgagaaacaaaacaaactagaaatagataaaaaaa    2561
LsMvp3      ctcgcgatgccgaaatcgagtatattgagaaacaaaacaaactagaaatagataaacaaa    2533
            *  * ** ** ** **  * ******** * *** ** ************** *** ***

LsMvp1      gtgagctgatgaagattgaagtggaaaaatttaatgctatggtagaggctgtaggaccag    2516
LsMvp2      tggaaataatgaggcttgaagtggaaaagtttaaggcaatggtgaatgctgttggacctc    2621
LsMvp3      cagaaataatgaggcttgaagtggaaaagtttaaggcaatggtaaatgctgttggacctc    2593
              **  * **** * ************* ***** ** *****  * ***** *****

LsMvp1      atacaattctatctatgtctgcactgccacgaaaacaccagattaaaatgttggagtcct    2576
LsMvp2      aaacacttctttccatgtcggctcttccaagagaacatcaaatccagatgttaaagtctt    2681
LsMvp3      aaacacttctttccatgtcggctcttccaagagagcatcaaatccagatgttaaagtctt    2653
            * *** **** ** ***** ** ** *** ** * ** ** **  * *****  **** *

LsMvp1      tgggtttgaaatctactctcatcaccgatggaaagactcctattaatttaattgatgctg    2636
LsMvp2      tgggcttgaagtcaacactcattacggacggtaaaacacccattaatttgattgatgctg    2741
LsMvp3      tgggcttgaagtcaacactcattacggatggtaaaacaccaattaatttgattgatgcag    2713
            **** ***** ** ** ***** ** ** ** ** ** ** ******** ******** *

LsMvp1      ccaagggacttgtggctgtgcatcaacaatcattctgaaactaatccaaatattgattag    2696
LsMvp2      cgaaagggctcattggagtcggtcaagaaactgtggcggaatgagaaaacatgattaaa-    2800
LsMvp3      caaaagggctcattggagtctgtcaagaaactgcagggaaatgagaaaatatgatataa-    2772
            * ** ** **  * *  **   **** ** *         * * *   **          *

LsMvp1      taattttatat-------caaataatagtgtcagtatatatcaaaagttgccatgacctg    2749
LsMvp2      aatttatacattgtcactgcgattgatatctcaggcatatttaattgttgcttttaccg-    2859
LsMvp3      aatttgtacatcgtcagtacgattgggatctccaacatat-----tgttgcttttaccg-    2826
             * ** ** **          **      * **              *****  * ***

LsMvp1      gatccagttacaatatatcat-------ctcattgaatttattcaccctttcttttta-t    2801
LsMvp2      atacttgtttatttatatactattcacagttatgagctatatgcttcatttttattatga    2919
LsMvp3      atacttgttgatttatatactgtttagacctaaatgct-------tcctattgattataa    2879
               *  ***    *****  *           *     *         * * **  **

LsMvp1      atatcaatatatctatcaaataaataaactcggttttctatcat----gaataa------    2851
LsMvp2      tttttatgcaatatgatacatttagcgattcacattatgattatttaagtctaatgatac    2979
LsMvp3      ttaata-------tgatatatttagcgatttaccttattat-----------------ac    2915
             *   *        *   * **  *   * *    **   **

LsMvp1      ----tgattaatatttaataaacatcctttagtggattaagctg----------------    2891
LsMvp2      tcagtttttgtgttttaatatgaagtatttaataaatacaattttacaa-----------    3029
LsMvp3      tccgtttttatattttaatattaagcatttaacaaatacatacaatgttggtactttatt    2975
                *  **    *******   *   ****     **  *

LsMvp1      --        2891
LsMvp2      --        3029
LsMvp3      gc        2977
```

Supplementary Figure S7: Primers used for *in situ* hybridization are marked in purple. For qPCR, the primers for LsMvp1, LsMvp2, and LsMvp3 are represented in yellow, green, and blue, respectively.

Figure 4c

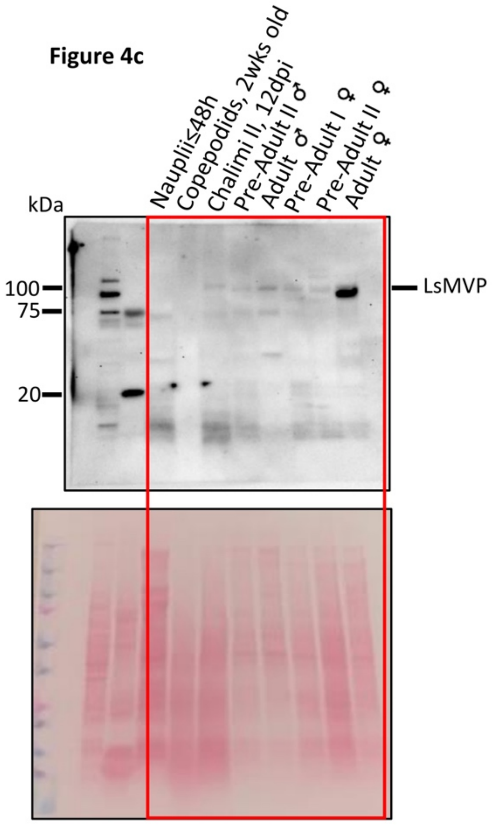

Figure 4d

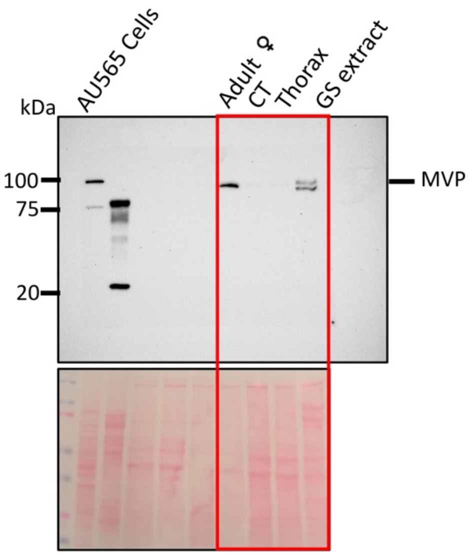

Figure 4e

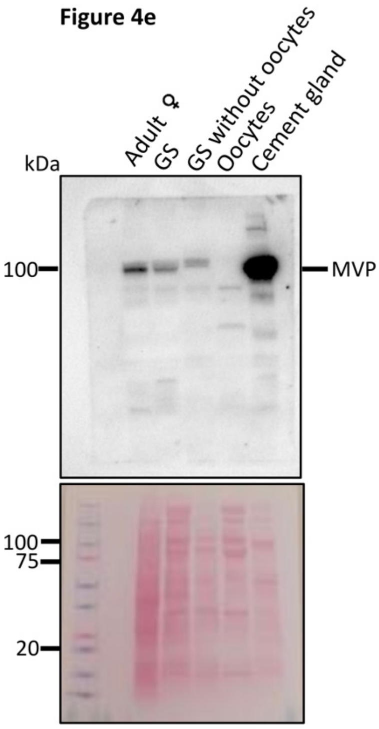

Figure 4f

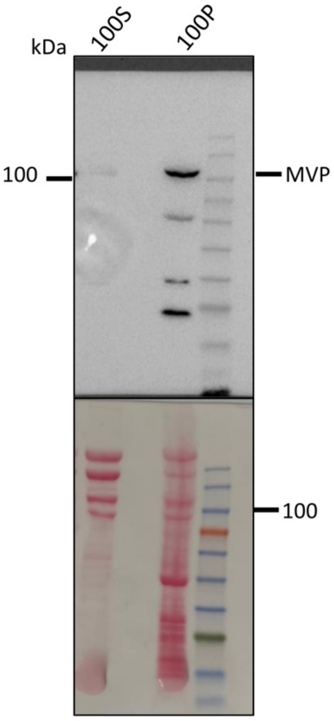

Figure 5b

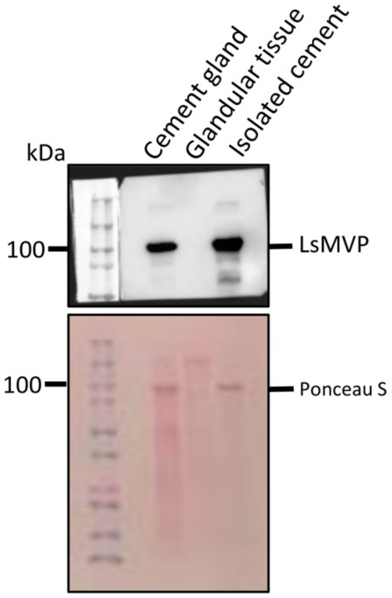

Figure 5d

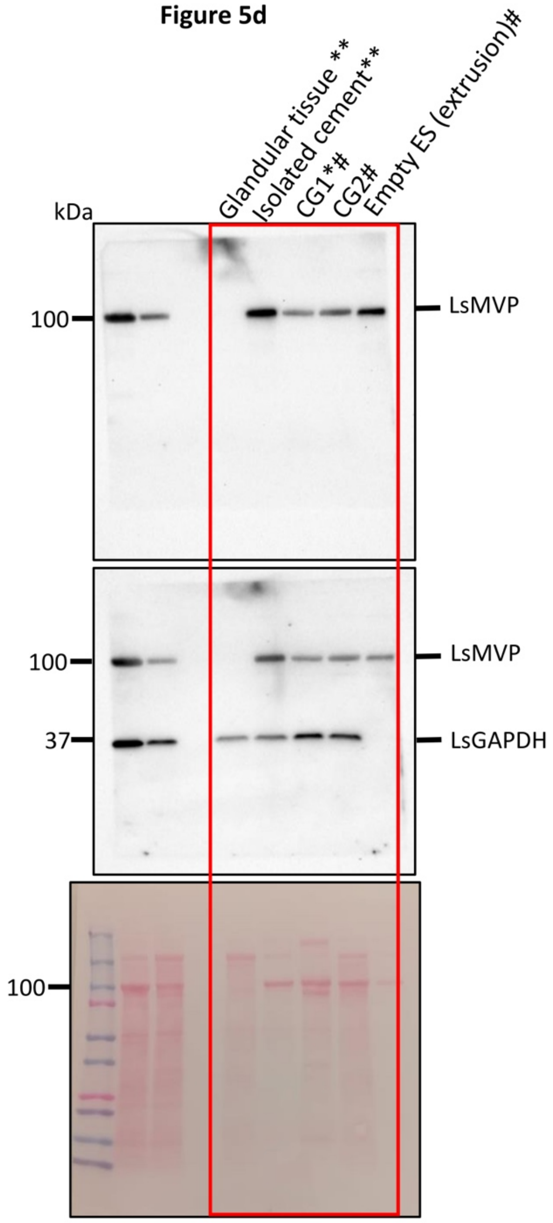

CG- cement gland
ES- Egg string
*- Gland where empty egg string was extruded from
#- from the same individual
**- from the same cement gland

Figure S1a

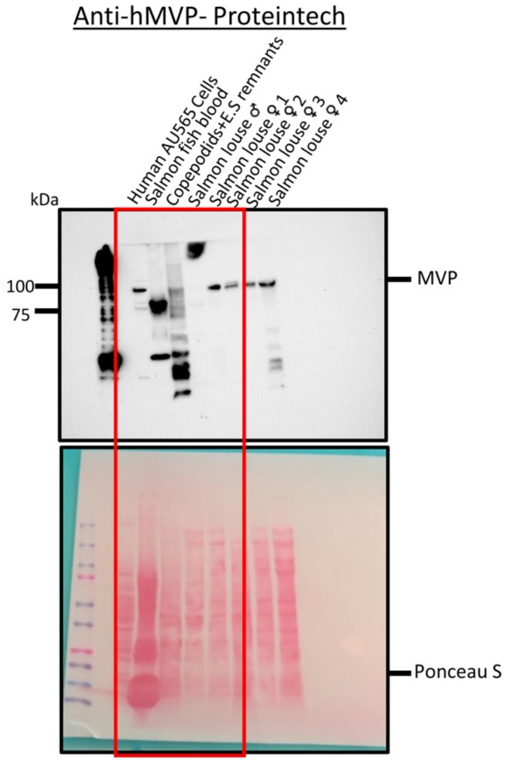

Figure S1b

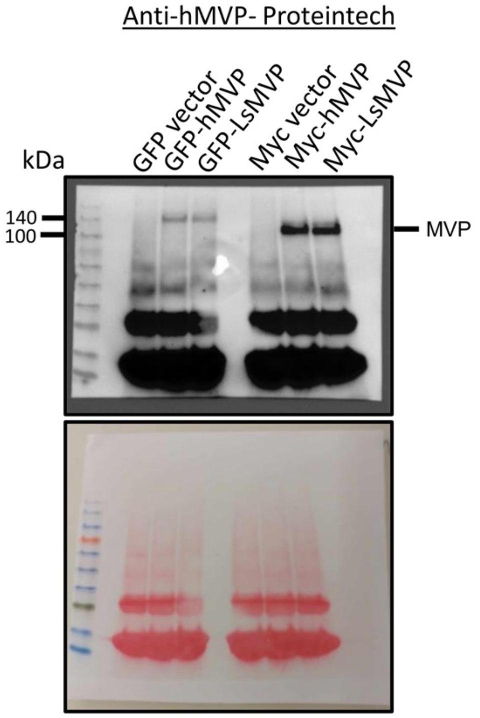

Figure S1c

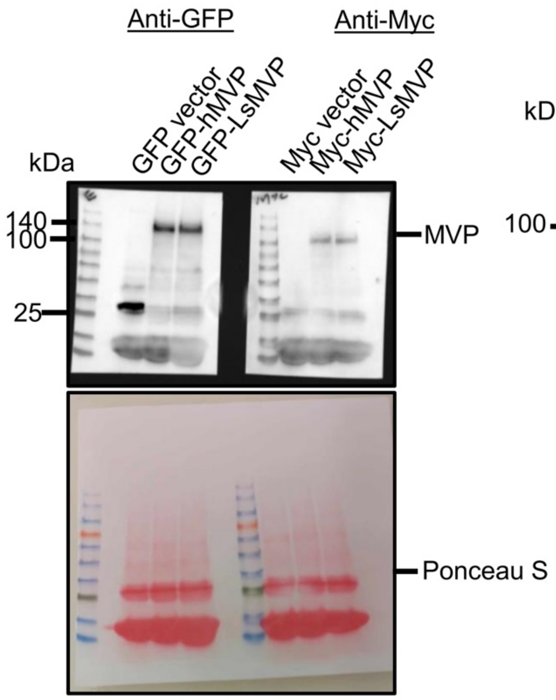

Figure S4

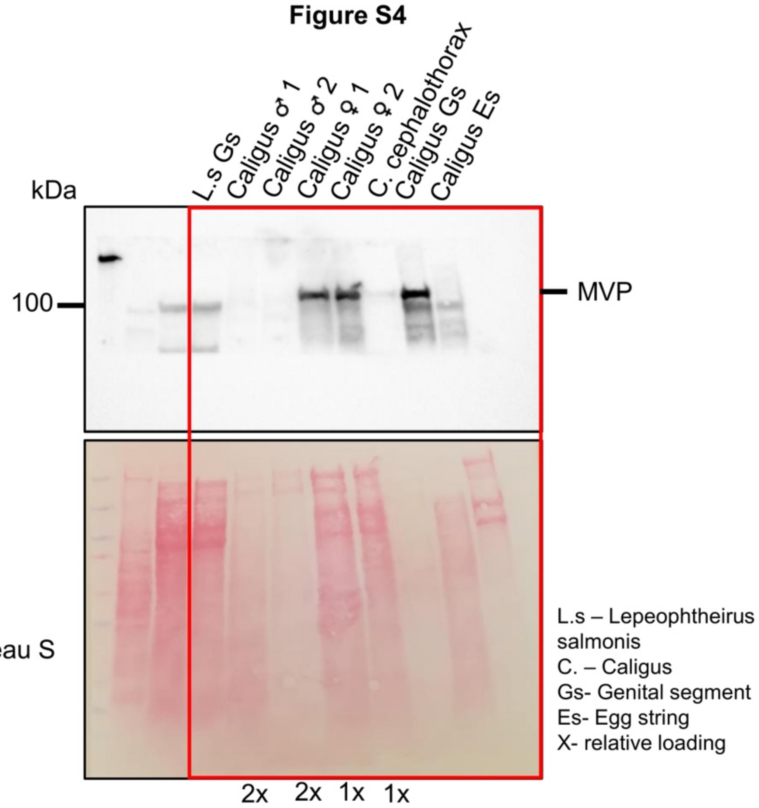

Supplementary Figure S8: Original blots for Figure 4 c-f, 5b and d, and Supplementary Figure S1 a-c and Figure S4, including ponceau S staining. Red squares show cropped regions used in the other figures.

Supplementary Tables

Supplementary Table S1: Primers used in this study and their application.

Name	Primer sequence 5` → 3`	Application
**LsMvp1_5R**	GGGCTCCAACATTACGAGATCAGGTCCATA	5`RACE
**LsMvp1_3R**	AAGTAAGAGGGGCCGTTTCATCCGTATCAT	3`RACE
**LsMvp2_5R**	GTGAATCTCGTGTTCGTTGATCAACAG	5`RACE
**LsMvp2_5RNested**	TCCATTCACCTCTATCTGCTGTAACATCCC	5`RACE
**LsMvp2_3R**	ACTGAGTGCTGCTCGTGAAACTTTA	3`RACE
**LsMvp2_3RNested**	TTGGTTAAGTACGAGGATAAGTCAGTCT	3`RACE
**LsMvp3_5R**	TCCAGGTTTTCGCATTTCTGGGGGAGTC	5`RACE
**LsMvp3_5RNested**	GATACAACGCAATAAGATCCTGGAGGG	5`RACE
**LsMvp3_3R**	TACATGCACGTCCTGGATCAAAACACTAAC	3`RACE
**LsTep1_5R**	AAGATCTTCGGGCTCGTGCAATTTGGGA	5`RACE
**LsTep1_3R**	GAACTCGGGGTACAAACATGGCATTGTG	3`RACE
**LsTep1_f_1**	CCACTAAAAATAGAATATTGACATCTACCA	Seq. LsTep1
**LsTep1_r_1**	CATATTGATAGGTGAGCATAGGGTTGTT	Seq. LsTep1
**LsTep1_f_nested1**	CAAAACCAGATCCTGCTCTACTTAA	Seq. LsTep1
**LsTep1_r_nested1**	CACGCATTGGTGATAAAAATACTCCATAGAA	Seq. LsTep1
**LsTep1_f_2**	TGTATCATGGAGTATTTGGAGCATC	Seq. LsTep1
**LsTep1_r_2**	GTCGGATTCCTTTAGTTTCCATATTTTG	Seq. LsTep1
**LsTep1_f_nested2**	CGTATTATTGTTTCGTCTAATACATCTGAC	Seq. LsTep1
**LsTep1_r_nested2**	GTGGAAGAAATGCTCGATAGAATCC	Seq. LsTep1
**M13_f**	GTAAAACGACGGCCAG	Cloning
**M13_r**	CAGGAAACAGCTATGAC	Cloning
**LsMvp_ISH_f**	GATCCTTTTCCATTATATCCAGG	*In situ* hyb.
**LsMvp_ISH_r**	CCATCCTCAAGAATTTCTCCTG	*In situ* hyb.
**LsMvp_ISH_T7_f**	TAATACGACTCACTATAGGGAGAGATCCTTTTCCATTATATCCAGG	*In situ* hyb.
**LsMvp_ISH_T7_r**	TAATACGACTCACTATAGGGAGACCATCCTCAAGAATTTCTCCTG	*In situ* hyb.
**LsMvp1_RNAi_f**	TAATACGACTCACTATAGGGagaCACATGGAGACGAAGAAGTGCGAT	RNAi
**LsMvp1_RNAi_r**	TAATACGACTCACTATAGGGagaGTGTGATAGCATCCACTGTGCCAA	RNAi
**LsMvp2_RNAi_f**	TAATACGACTCACTATAGGGagaCCTCCCACCCGAATGGTCGTA	RNAi
**LsMvp2_RNAi_r**	TAATACGACTCACTATAGGGagaCCTCCCCTGCCACTCTCTTACAAC	RNAi

Supplementary Table S2: Primers, Tm, efficiency, and product size for qPCR assays.

Name	Primer sequence 5`→ 3`	Tm (°C)	Efficiency	Product size (bp)
**LsMvp1_f**	AAGGGAAGGCAAAGCTTGGACAT	69.6	2.06	187 bp
**LsMvp1_r**	GGATTTCTGACGATTGAAGAGCCTG	69.6		
**LsMvp2_f**	TCGGTCAAGAAACTGTGGC	64.0	2.04	150 bp
**LsMvp2_r**	AATGAAGCATATAGCTCATAACTGTG	61.3		
**LsMvp3_f**	CTGTCAAGAAACTGCAGGGA	63.1	2.00	140 bp
**LsMvp3_r**	CAATAGGAAGCATTTAGGTCTAAACAG	62.9		
**LsEF1α_f**	GGTCGACAGACGTACTGGTAAATCC	67.4	1.99	229 bp
**LsEF1α_r**	TGCGGCCTTGGTGGTGGTTC	74.4		
**LsADT3_f**	CTGGAGAGGGAATTTGGCTAACGTG	71.0	1.94	302 bp
**LsADT3_r**	GACCCTGGACACCGTCAGACTTCA	72.6		

Supplementary Table S3: Number of salmon lice recovered from 30 lice of each group distributed on 3 fish each.

	Control (dsCYP)	Knock-down (dsMVP)	Trypan
**Experiment 1**	17 (6,6,5)	15 (4,4,7)	
**Experiment 2**	17 (7,5,5)	15 (3,8,4)	18 (5,5,8)
